# Role of Epoxide Functionalization of Amines for Development of Direct Air Capture Sorbents with High Cyclic Working Capacity at Low Desorption Temperatures

**DOI:** 10.1002/advs.75091

**Published:** 2026-04-02

**Authors:** Joo Yeon Han, Hayoung Jeong, Younghyu Ko, Yohan Cho, Seenu Ravi, Kyu‐Min Ryoum, Hyug Hee Han, Yujin Choi, Chaewon Shin, Jeong Woo Han, Youn‐Sang Bae

**Affiliations:** ^1^ Department of Chemical and Biomolecular Engineering Yonsei University Seoul South Korea; ^2^ Department of Materials Science and Engineering Research Institute of Advanced Materials Seoul National University Seoul South Korea

**Keywords:** amine‐impregnated mesoporous silica, butylene oxide, CO_2_ adsorbent, desorption temperature, direct air capture, molecular dynamics simulation

## Abstract

Broad implementation of the direct air capture (DAC) technology requires sorbents that can achieve high cyclic CO_2_ working capacities (WC_cyclic_) at low desorption temperatures. This study shows that appropriate degrees of butylene oxide (BO) functionalization on amines with different molecular weights (polyethyleneimine (PEI1200 and PEI300) and tris(2‐aminoethyl)amine (TREN)) can achieve excellent WC_cyclic_ at low desorption temperatures (40–70°C). Through systematic screening, 0.30BO‐PEI300‐SY and 0.54BO‐TREN‐SY are identified as the optimal sorbents with the highest WC_cyclic_ at desorption temperatures of 45 and 40°C, respectively. Both sorbents exhibit excellent stability under oxygen‐rich and humid conditions, maintaining outstanding WC_cyclic_ compared to other benchmark DAC materials. Molecular dynamics simulations reveal that CO_2_ adsorption on 1° amine sites plays a dominant role in determining the overall CO_2_ capture capacity of pristine amines, and the reduction in CO_2_ uptake after BO treatment is primarily attributable to the loss of accessible 1° amine sites. Both experimental and simulation results highlight that the fraction of 1° amines is a key factor governing WC_cyclic_ and desorption behavior after BO modification.

## Graphical abstract

Appropriate butylene oxide (BO) functionalization of amines (polyethyleneimine (PEI1200 and PEI300) and tris(2‐aminoethyl)amine (TREN)) enables excellent cyclic working capacities (WC_cyclic_) at low desorption temperatures (40−70°C). Notably, 0.30BO‐PEI300‐SY and 0.54BO‐TREN‐SY show the highest WC_cyclic_ at 45 and 40°C, respectively. Experiments and simulations reveal that the fraction of primary amines governs WC_cyclic_ and desorption behavior after BO modification.

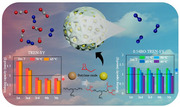

## 1 Introduction

Global warming remains one of the most urgent challenges facing future generations. Global CO_2_ emissions reached 37.4 Gt in 2023, and atmospheric CO_2_ concentrations surpassed 420 ppm in 2024, which is 47% higher than the pre‐industrial levels [1]. To limit global temperature rise to below 2°C by 2100, an estimated 50 Gt of CO_2_ must be removed annually [2]. While nature‐based solutions such as bioenergy with carbon capture and storage and afforestation have been explored, their substantial land requirements and emissions from biomass supply chains raise concerns about sustainability and cost‐effectiveness. [3, 4, 5]

Conventional carbon capture technologies primarily target high‐concentration point sources such as fossil fuel power plants. However, nearly half of global CO_2_ emissions come from diffuse sources such as residential and commercial buildings [6, 7, 8]. Direct air capture (DAC), which removes CO_2_ directly from ambient air, is a promising alternative due to its flexible siting and minimal land use. While the current DAC capacity remains low (∼0.01 Mt/year), increasing governmental support and ongoing technological advancements are enhancing its scalability and economic viability [9, 10].

The most advanced DAC technologies today are categorized into liquid solvent‐based and solid sorbent‐based systems. The liquid systems, exemplified by Carbon Engineering (Canada), have been demonstrated at the scale of several thousand tons [11]. However, the high temperature required by these systems (∼900°C) increases the energy consumption, operating costs, and water use [12]. As a result, solid sorbent‐based approaches are gaining attraction [11, 13]. Compared to the liquid systems, solid sorbents typically generate less waste and require less energy to regenerate [11, 14]. Recently, moisture swing adsorption, which minimizes the desorption energy consumption by modulating humidity rather than increasing temperature, has also attracted significant attention. Although this strategy offers substantial energy savings compared to conventional regeneration methods, precise humidity control necessitates complex process design. Moreover, it often suffers from slower kinetics and lower adsorption capacities, and its performance is highly dependent on climatic and geographic conditions. Consequently, temperature swing adsorption (TSA) remains the most widely adopted regeneration approach in industrial applications [15, 16, 17]. The primary candidates most widely employed in TSA processes are amine‐incorporated sorbents. Climeworks (Switzerland) has commercialized DAC systems using such sorbents, operating plants in locations such as Iceland. Nonetheless, these systems still require thermal or vacuum regeneration at 80–100°C, making them economically viable only in regions with access to low‐cost renewable heat, such as geothermal energy [18]. Moreover, the sorbent durability and performance under humid conditions still need improvement. To enable broader deployment of DAC, it is necessary to develop sorbents with high humidity resistance, long‐term stability, and efficient low‐temperature regeneration [8, 19].

Given the extremely low CO_2_ concentration in ambient air, amine‐incorporated sorbents are preferred due to their stronger binding affinity, high stability under humid conditions, and superior performance [20]. Among various properties of amine molecules, such as structure, molecular weight, and viscosity, the ratio of primary (1°), secondary (2°), and tertiary (3°) amines (1°:2°:3°) is particularly critical, as it governs adsorption performance through the different interaction energies with CO_2_ [21, 22, 23, 24]. Typically, 1° amines form ammonium carbamates via a reaction involving two 1° or 2° amines per CO_2_ molecule, while 2° amines form carbamic acids, and both products contribute significantly to CO_2_ capture. In contrast, 3° amines exhibit weaker interactions, forming unstable ammonium carbamates with poor kinetics. Thus, the amine type ratio is expected to significantly influence CO_2_ adsorption–desorption behavior [25, 26]. Choi et al. demonstrated that treating polyethylenimine (PEI) with epoxides can convert 1° amines into 2° or 3° amines, thereby suppressing urea formation and oxidative degradation [27]. Epoxide‐functionalized PEI also showed improved desorption efficiency and reduced heat of regeneration. However, these findings are limited to flue gas applications under CO_2_‐rich desorption conditions, which differ significantly from DAC systems that operate with N_2_ or air during desorption. A more recent study explored epoxide functionalization of various amines to address volatility in low‐molecular‐weight species and improve thermal stability at elevated temperatures [28]. Nevertheless, no systematic study has been conducted on how epoxide functionalization affects CO_2_ adsorption–desorption performance under temperature swing adsorption conditions, which are relevant to DAC.

This study focuses on two key performance metrics of DAC sorbents: cyclic CO_2_ working capacity (WC_cyclic_) and desorption temperature. WC_cyclic_ refers to the amount of CO_2_ a sorbent can repeatedly capture under defined adsorption–desorption cycles. While conventional amine‐based DAC sorbents often demonstrate high WC_cyclic_, they typically require high desorption temperatures (≥80–150°C), which limits their practical applicability. To facilitate broader deployment of DAC technologies, it is essential to develop sorbents that maintain sufficient working capacities at lower desorption temperatures.

**SCHEME 1:**
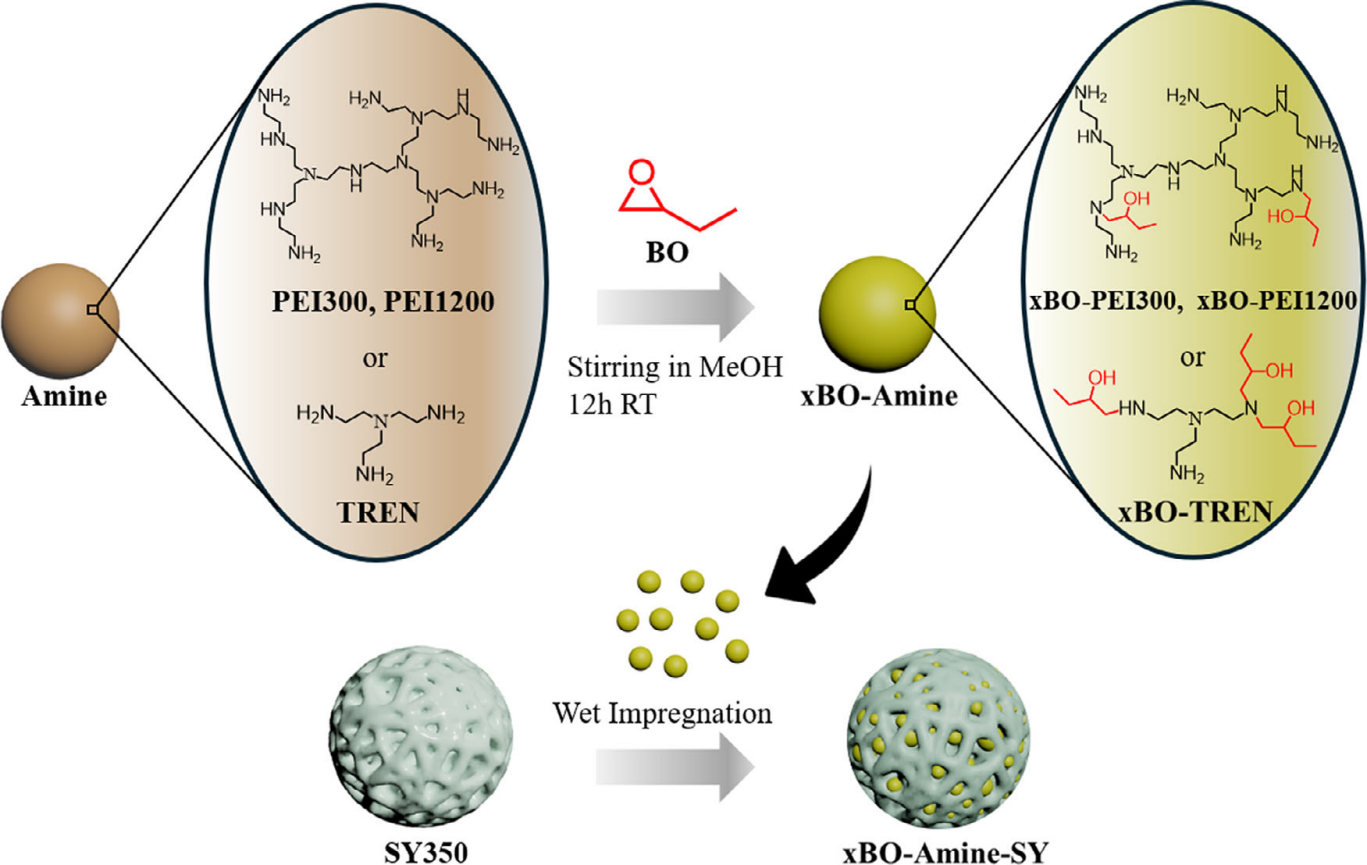
Schematic syntheses of xBO‐Amine and xBO‐Amine‐SY (Amine = PEI1200, PEI300, or TREN).

We hypothesized that functionalizing amines with epoxide, which partially converts 1° amines into 2° or 3° amines, could reduce the desorption temperature, albeit at the expense of WC_cyclic_. To validate this hypothesis, we used butylene oxide (BO) to functionalize three structurally distinct amines with varying molecular weights and amine compositions, namely PEI1200, PEI300, and tris(2‐aminoethyl)amine (TREN) (Scheme 1). BO was selected as the functionalizing agent based on a prior report demonstrating its superior moisture resistance and lower regeneration energy compared to other epoxides [29]. Unlike many previous studies evaluating DAC sorbents under idealized conditions (involving complete adsorption and desorption), here we assessed WC_cyclic_ under more realistic time constraints of 180 min for adsorption and 90 min for desorption. Sylysia 350 (SY350), a commercially available mesoporous silica with large pore sizes, was used as the support due to its favorable textural properties and low cost. To complement the experimental analysis, molecular dynamics (MD) simulations were also performed to provide molecular‐level insights into how BO functionalization affects CO_2_ adsorption behavior.

## 2 Results and Discussion

### 2.1 Characterization of xBO‐Amine and xBO‐Amine‐SY

To determine the 1°:2°:3° amine ratios of PEI1200, PEI300, TREN, and their BO‐functionalized derivatives, ^13^C NMR spectra were obtained (Figures S3–S5). The amine ratios were calculated using the integrated NMR peaks and summarized in Figure S1. The ratios of pristine PEI1200, PEI300, and TREN are 39:35:26, 44:41:15, and 75:0:25, respectively. The ratios of PEI1200 and PEI300 are consistent with those reported in previous studies [30, 31]. In the case of TREN, the calculated ratio aligns well with its molecular structure consisting of three 1° amines bonded to a central 3° amine. As the degree of BO functionalization increased, the proportion of 1° amines in PEI1200 decreased gradually from 39% to 14%, while those of 2° and 3° amines increased from 35% to 53% and from 26% to 33%, respectively. A similar conversion pattern was observed in PEI300. TREN was functionalized with a larger amount of BO (≥0.36 BO) compared to the PEIs for two reasons: (i) to prevent volatilization during the desorption process, owing to its lower molecular weight, and (ii) to reduce the high desorption energy resulting from its high 1° amine content. For all three amine types, BO functionalization decreased the proportion of 1° amines and increased those of 2° and 3° amines. However, the overall conversion from 2° to 3° amines was lower than the conversion from 1° to 2°, likely due to the higher nucleophilicity and reactivity of 1° amines toward epoxide groups [27, 32, 33]. Since 3° amines have a significantly lower affinity for CO_2_, a selective conversion of 1° to 2° amines may be the optimal strategy to achieve a balance between moderate adsorption capacity and low desorption temperature.

The pristine amines (referred to as “Amines” below) and BO‐functionalized amines (referred to as “xBO‐Amines”) were impregnated into the mesopores of SY350 (SY) using a fixed 1:1 mass ratio to ensure consistency across all samples. Here, x represents the molar ratio of BO to nitrogen atoms (O/N ratio) in the amine. To investigate the textural properties of SY and the amine‐impregnated materials (referred to as Amine‐SY and xBO‐Amine‐SY), N_2_ adsorption–desorption isotherms were measured at 77 K (Figure S6). Pristine SY exhibited a typical type IV isotherm (Figure S6a), indicative of a mesoporous structure, with a broad pore size distribution ranging from 7 to 43 nm (Figure S7a) [34]. Its Brunauer–Emmett–Teller (BET) surface area (S_BET_) and total pore volume (V_total_) were 294.8 m^2^ g^−1^ and 1.63 cm^3^ g^−1^, respectively (Table S1). Despite its relatively lower S_BET_, the pore size and V_total_ of SY are larger than those of well‐known mesoporous silica materials, for example, MCM‐41 (pore size = 3–6 nm, V_total_ = 0.7–1.2 cm^3^ g^−1^, S_BET_ = 1000–1050 m^2^ g^−1^) [35, 36] and SBA‐15 (pore size = 9–12 nm, V_total_ = 0.4–1.4 cm^3^ g^−1^, S_BET_ = 400–850 m^2^ g^−1^) [37, 38]. Larger pore size and total pore volume are advantageous for CO_2_ sorbent supports because they promote diffusion and minimize pore blockage during amine impregnation, as confirmed by CO_2_ adsorption experiments conducted on three different PEI1200‐impregnated mesoporous silicas (Figure S30b) [39, 40, 41, 42]. Given its large pore volume and pore size, SY350 was considered highly suitable for amine impregnation [43, 44, 45]. As expected, impregnating SY with pristine amines resulted in significant reductions in S_BET_ and V_total_ (Table S1). Due to the identical 1:1 mass ratio applied to all samples, the three amine‐loaded SYs showed similar S_BET_ (54.3–58.4 m^2^ g^−1^) and V_total_ (0.35–0.38 cm^3^ g^−1^). Furthermore, when BO‐functionalized amines were impregnated into SY, even greater decreases in S_BET_ and V_total_ were observed. The extent of this reduction increased with the amount of bulky BO moieties, indicating that BO functionalization further hinders porosity by introducing steric effects.

### 2.2 Determining the Cyclic CO_2_ Working Capacity and Desorption Temperature

**FIGURE 1:**
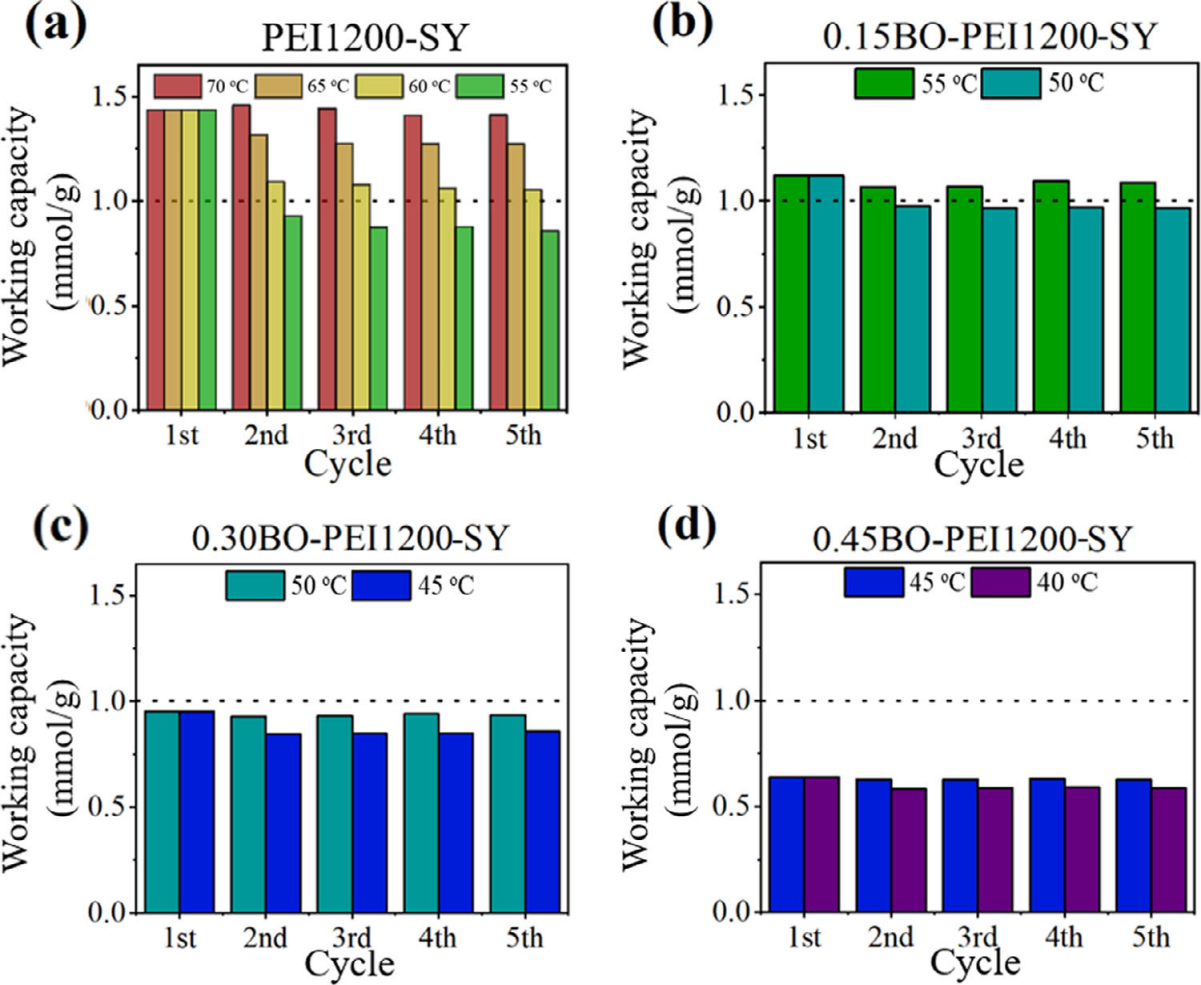
CO_2_ working capacities of (a) PEI1200‐SY, (b) 0.15BO‐PEI1200‐SY, (c) 0.30BO‐PEI1200‐SY, and (d) 0.45BO‐PEI1200‐SY over five adsorption–desorption TGA cycles under various desorption temperatures.

**FIGURE 2:**
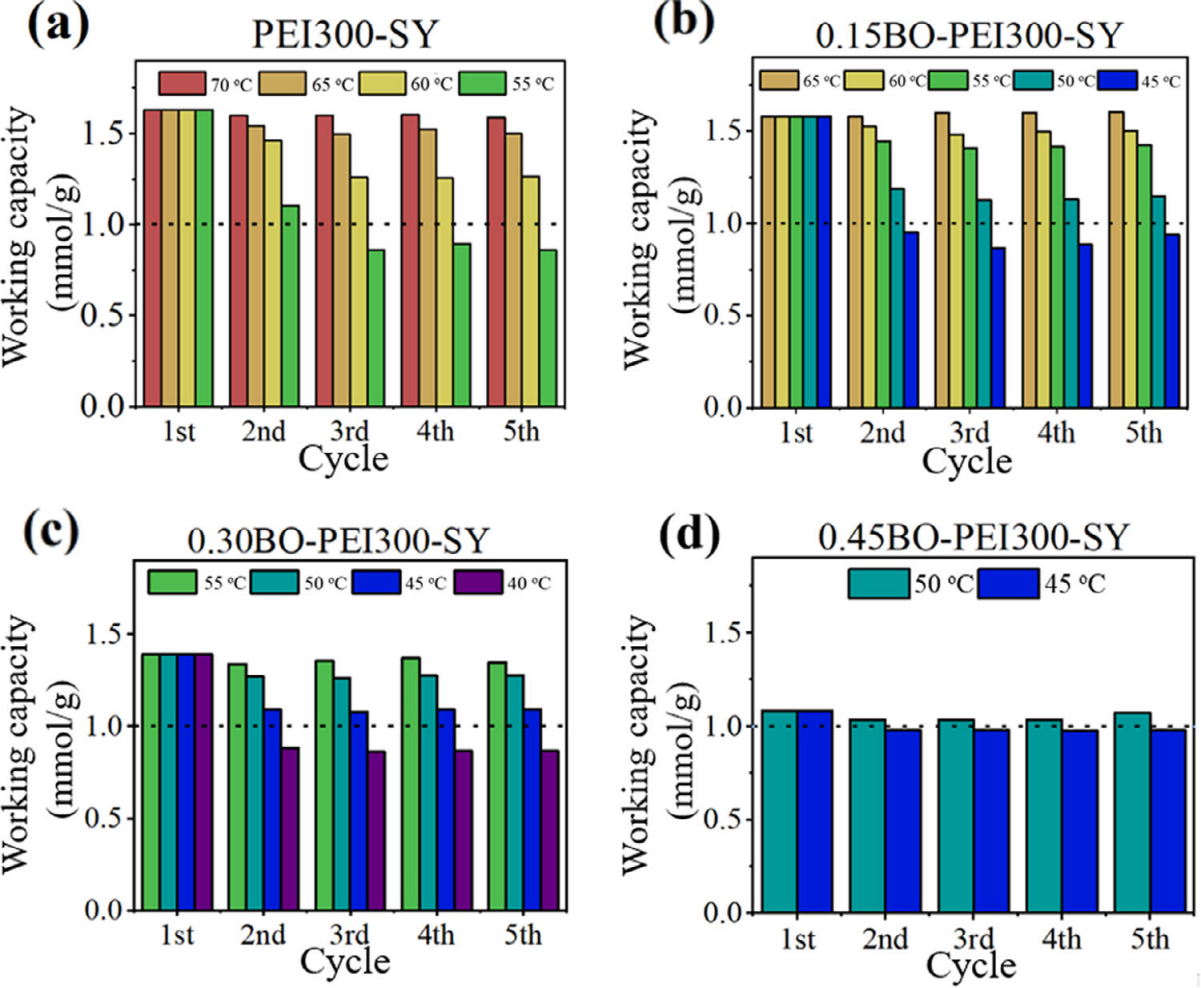
CO_2_ working capacities of (a) PEI300‐SY, (b) 0.15BO‐PEI300‐SY, (c) 0.30BO‐PEI300‐SY, and (d) 0.45BO‐PEI300‐SY over five adsorption–desorption TGA cycles under various desorption temperatures.

**FIGURE 3:**
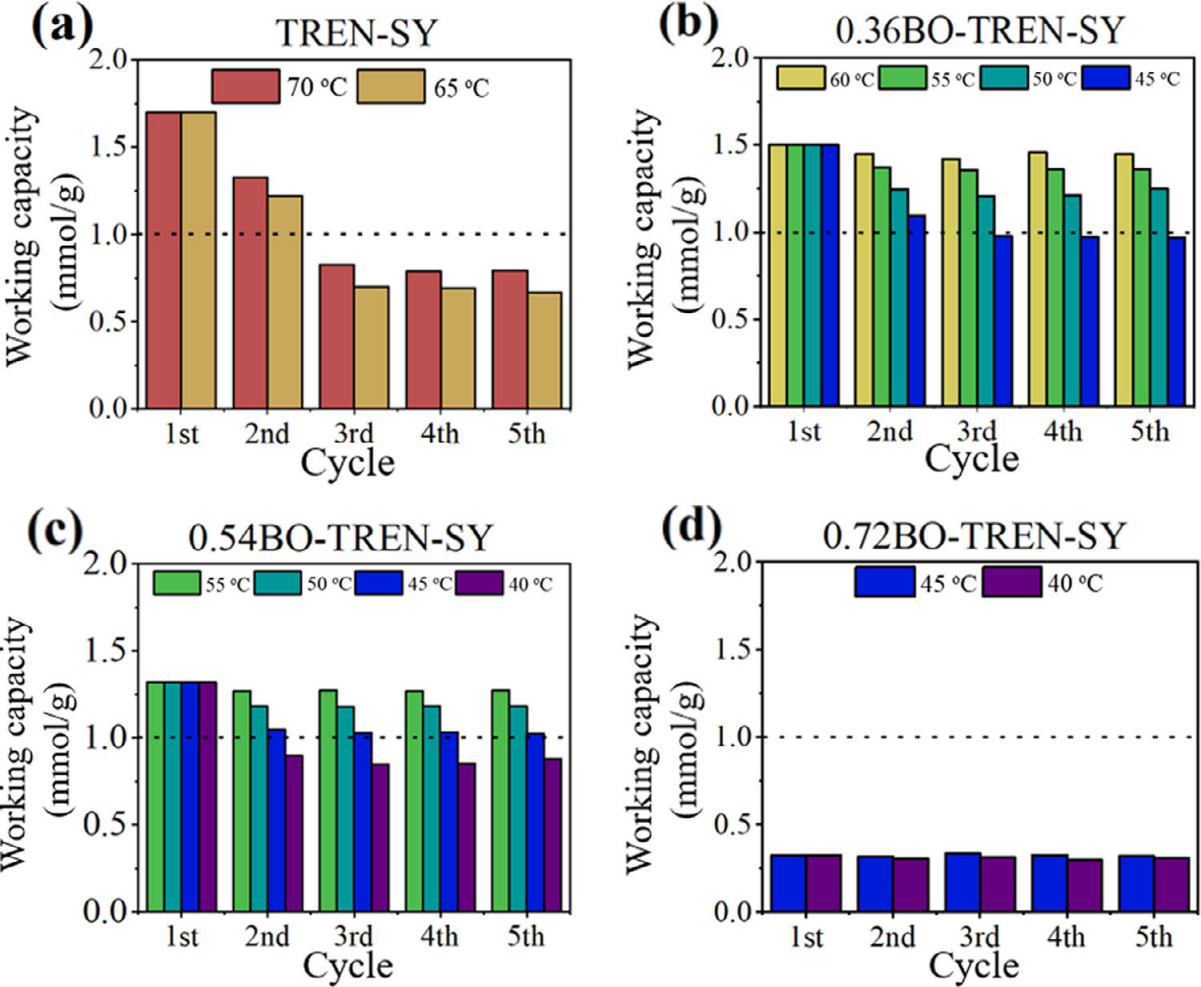
CO_2_ working capacities of (a) TREN‐SY, (b) 0.36BO‐TREN‐SY, (c) 0.54BO‐TREN‐SY, and (d) 0.72BO‐TREN‐SY over five adsorption–desorption TGA cycles under various desorption temperatures.

To investigate the effect of BO functionalization of amines on their WC_cyclic_ values and desorption temperature, TGA experiments were conducted over five adsorption–desorption cycles (Figures S8–S19). For all materials, the adsorption and desorption durations were fixed at 180 and 90 min, respectively. Adsorption was performed at a constant temperature of 25°C, while the desorption temperature was systematically varied. CO_2_ uptake was calculated based on the weight gain of the adsorbent, assuming that N_2_ uptake was negligible [46]. The CO_2_ working capacity of each sample was defined as the weight gain during the adsorption step of each cycle. To efficiently evaluate desorption behavior, we defined the baseline desorption temperature (T_base‐des_) as the minimum temperature at which the working capacity decreased to less than 5% of its initial value after five adsorption–desorption cycles. For example, T_base‐des_ of PEI1200‐SY was determined to be 70°C, because the working capacity decreased by only 1.8% after five cycles at this temperature (Figure 1a). In contrast, at a desorption temperature of 65°C, the working capacity dropped by 11.5%. Using the same criterion, we determined the T_base‐des_ values of xBO‐PEI1200‐SY samples. As the BO loading increased, T_base‐des_ continuously decreased, reaching as low as 45°C for 0.45BO‐PEI1200‐SY (Figure 1b–d). This confirms our hypothesis that BO functionalization reduces the proportion of 1° amines, thereby enabling desorption at lower temperatures. Similarly, PEI300‐SY exhibited a T_base‐des_ of 70°C, which progressively decreased to 50°C with increasing BO content in the xBO‐PEI300‐SY samples (Figure 2). However, it was impossible to determine T_base‐des_ for TREN‐SY using the same criterion. Since TREN contains a much higher proportion of 1° amines than PEI (75% vs. 39% for PEI1200 and 44% for PEI300), TREN‐SY showed a significant loss in working capacity over five cycles at 70°C. Nevertheless, desorption at temperatures above 70°C was not feasible due to the low molecular weight of TREN and the resulting volatility. Therefore, 70°C was adopted as the T_base‐des_ for TREN‐SY (Figure 3a). In contrast, T_base‐des_ for xBO‐TREN‐SY could be determined at temperatures below 70°C due to their reduced 1° amine content. Additionally, the introduction of bulky BO groups helped suppress volatility. Similar to the PEI‐based samples, increasing the BO content led to a progressive decrease in T_base‐des_ of xBO‐TREN‐SY, reaching as low as 45°C (Figure 3b–d).

After determining the T_base‐des_ for each sample, additional TGA experiments were conducted for five adsorption–desorption cycles, in which the desorption temperature was reduced by 5°C below T_base‐des_. In all cases except TREN‐SY, the CO_2_ working capacity decreased between the first and second cycles but remained nearly constant from the second to the fifth cycle. In contrast, TREN‐SY showed a significant decline in working capacity from the first to the third cycle, after which it stabilized through the fifth cycle (Figure 3a). Since the working capacity was consistently maintained during cycles 3–5 for all samples, the CO_2_ working capacity measured in the fifth cycle was defined as the cyclic working capacity (WC_cyclic_). Although WC_cyclic_ decreased when desorption was carried out at 5°C below T_base‐des_, its stability after the third cycle indicated that the sorbents remained usable even at lower desorption temperatures.

We assumed that a WC_cyclic_ value near 1 mmol g^−1^ is necessary for practical DAC applications [47]. Therefore, we did not perform further experiments at lower desorption temperatures for samples with a WC_cyclic_ less than 1 mmol g^−1^ at 5°C below T_base‐des_ (Figures 1, 2, 3). These samples were 0.15BO‐PEI1200‐SY, 0.30BO‐PEI1200‐SY, 0.45BO‐PEI1200‐SY, 0.45BO‐PEI300‐SY, TREN‐SY, and 0.72BO‐TREN‐SY. The remaining samples were tested with the desorption temperature lowered in 5°C increments until WC_cyclic_ dropped below 1 mmol g^−1^.

### 2.3 Effect of BO‐Functionalization of Amines on Cyclic CO_2_ Working Capacity and Desorption Temperature

**FIGURE 4:**
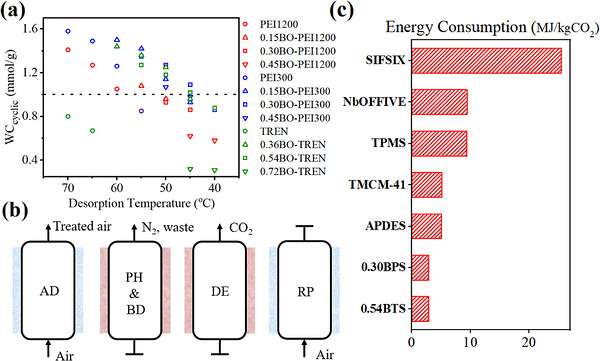
(a) WC_cyclic_ of Amine‐SY and xBO‐Amine‐SY materials at various desorption temperatures. For clarity, “‐SY” has been omitted from the material names. Red: xBO‐PEI1200‐SY, blue: xBO‐PEI300‐SY, green: xBO‐TREN‐SY. (b) The four steps of the TVSA process in DAC: AD (adsorption), PH (pre‐heating) & BD (blowdown), DE (desorption), and RP (re‐pressurization). (c) Comparison of the thermal energy consumption required for adsorption‐desorption cycles in the TVSA process for our materials (0.30BPS = 0.30BO‐PEI300‐SY; 0.54BTS = 0.54BO‐TREN‐SY) and previously reported benchmark chemisorbents and physisorbents for DAC.

First, we examine the TGA results of the samples impregnated by amines without BO treatment (Amine‐SY). At a desorption temperature of 70°C, WC_cyclic_ followed the order PEI300‐SY > PEI1200‐SY > TREN‐SY. This can be explained as follows. In the first cycle, the CO_2_ working capacity was in the order TREN‐SY > PEI300‐SY > PEI1200‐SY (Figures 1a, 2a, and 3a), consistent with the trend in 1° amine content (Figure S1) [48]. However, over five cycles, TREN‐SY exhibited a significant decrease in WC_cyclic_ due to overly strong adsorption and poor desorption at 70°C (Figure 3a), whereas PEI1200‐SY and PEI300‐SY showed only minor decreases in working capacity (by 0.86% and 2.6%, respectively, Figures 1a and 2a). As the desorption temperature decreased stepwise to 65°C, 60°C, and 55°C, the WC_cyclic_ of all Amine‐SY samples declined as expected, while their relative order remained unchanged (Figure 4). At 55°C, the WC_cyclic_ of both PEI300‐SY and PEI1200‐SY dropped below 1 mmol g^−1^, and hence no further experiments were performed at lower temperatures.

Next, we consider the BO‐treated PEI1200 samples (xBO‐PEI1200‐SY). For 0.15BO‐PEI1200‐SY, the T_base‐des_ was determined at 55°C, significantly lower than that of PEI1200‐SY. However, due to the reduced 1° amine content and lower desorption temperature, the WC_cyclic_ of 0.15BO‐PEI1200‐SY at T_base‐des_ (55°C) was lower than that of PEI1200‐SY at T_base‐des_ (70°C) (1.41 → 1.08 mmol g^−1^) (Figure 1a,b). At 50°C, the WC_cyclic_ fell below 1 mmol g^−1^ (Figure 1b), and hence no further experiments were conducted. Notably, at the same desorption temperature of 55°C, 0.15BO‐PEI1200‐SY showed a much higher WC_cyclic_ than PEI1200‐SY, indicating that an appropriate level of BO treatment can significantly lower the desorption temperature while maintaining a high WC_cyclic_. This behavior arises because the reduction in 1° amine content induced by BO treatment leads to two opposing effects. First, the decrease in the number of strong adsorption sites lowers the overall CO_2_ uptake. Second, the weakened CO_2_ binding strength facilitates desorption at lower temperatures, leading to a decrease in the amount of undesorbed CO_2_. The resulting WC_cyclic_ value is determined by the balance between these two effects. In the case of BO treatment from PEI1200‐SY to 0.15BO‐PEI1200‐SY, the enhancement in CO_2_ desorption at 55°C is significantly greater than the reduction in CO_2_ uptake (Figure S8d vs. Figure S9a). Consequently, 0.15BO‐PEI1200‐SY exhibits a considerably higher WC_cyclic_ value.

For 0.30BO‐PEI1200‐SY, the T_base‐des_ was 50°C, slightly lower than that of 0.15BO‐PEI1200‐SY, and the WC_cyclic_ was 0.93 mmol g^−1^ and further decreased to 0.86 mmol g^−1^ at 45°C (Figure 1c). For 0.45BO‐PEI1200‐SY, the T_base‐des_ was 45°C, but the WC_cyclic_ was only 0.62 mmol g^−1^ (Figure 1d). These results suggest that excessive BO treatment can further reduce desorption temperature but at the expense of a drastic loss in WC_cyclic_.

The BO‐treated PEI300 samples (xBO‐PEI300‐SY) showed similar trends to xBO‐PEI1200‐SY. However, owing to their higher proportion of 1° (or 1° + 2°) amines, they exhibited much higher WC_cyclic_ values (Figure 2b–d). For 0.15BO‐PEI300‐SY, the T_base‐des_ was determined at 65°C, 5°C lower than that of PEI300‐SY. Interestingly, even at this lower desorption temperature, its WC_cyclic_ slightly exceeded that of PEI300‐SY at 70°C (1.58 → 1.60 mmol g^−1^, Figure 2a,b). This can be explained by the balance between a slight reduction in adsorption capacity (due to decreased 1° amine content from 44% to 33%) and improved desorption efficiency, which resulted in smaller losses in working capacity over five cycles. As the desorption temperature decreased to 60°C, 55°C, 50°C, and 45°C, the WC_cyclic_ values declined to 1.50, 1.42, 1.14, and 0.93 mmol g^−1^, respectively (Figure 2b). For 0.30BO‐PEI300‐SY, the T_base‐des_ was 55°C, with a WC_cyclic_ of 1.35 mmol g^−1^, which further decreased to 1.27, 1.09, and 0.86 mmol g^−1^ at 50°C, 45°C, and 40°C, respectively (Figure 2c). For 0.45BO‐PEI300‐SY, the T_base‐des_ was 50°C, with a WC_cyclic_ of 1.07 mmol g^−1^, which decreased to 0.98 mmol g^−1^ at 45°C (Figure 2d). Interestingly, at certain desorption temperatures, samples with higher BO loadings exhibited higher WC_cyclic_ values than those with lower BO loadings. For example, at 55°C 0.15BO‐PEI300‐SY showed higher WC_cyclic_ than 0.30BO‐PEI300‐SY, but at 50°C the order was reversed. This behavior can likewise be explained by the two opposing effects arising from the BO‐induced reduction in 1° content, as discussed for PEI1200‐SY and 0.15BO‐PEI1200‐SY. Specifically, in the case of BO treatment from 0.15BO‐PEI300‐SY to 0.30BO‐PEI300‐SY, the reduction in CO_2_ uptake is slightly greater than the enhancement in CO_2_ desorption at 55°C (Figure S13c vs. Figure S14a). Consequently, 0.15BO‐PEI300‐SY exhibits a slightly higher WC_cyclic_ value than 0.30BO‐PEI300‐SY at this desorption temperature. In contrast, when the desorption temperature is lowered to 50°C, the enhancement in CO_2_ desorption becomes greater than the reduction in CO_2_ uptake for the same 0.15BO → 0.30BO treatment (Figure S13d vs. Figure S14b). As a result, 0.30BO‐PEI300‐SY exhibits a higher WC_cyclic_ value than 0.15BO‐PEI300‐SY at a desorption temperature of 50°C.

As noted earlier, to compensate for the volatility and excessively high 1° amine content of TREN, larger amounts of BO were introduced into xBO‐TREN‐SY than into xBO‐PEI1200‐SY or xBO‐PEI300‐SY. When comparing the adsorption capacities after the first cycle, a clear decreasing trend was observed with increasing degrees of BO treatment. However, over five cycles, pristine TREN‐SY exhibited very low WC_cyclic_ values of 0.6–0.7 mmol g^−1^ at 70°C and 65°C due to the combined effects of a sharp increase in irreversibly adsorbed CO_2_ resulting from the excessively high fraction of 1° amines and volatilization caused by the low molecular weight of TREN (Figure 3a and Figure S16). In contrast, 0.36BO‐TREN‐SY showed a T_base‐des_ of 60°C with a remarkably high WC_cyclic_ of 1.44 mmol g^−1^, which gradually decreased to 1.36, 1.25, and 0.96 mmol g^−1^ at 55°C, 50°C, and 45°C, respectively (Figure 3b). This improved performance can be attributed to BO functionalization, which increases the molecular weight and thereby reduces volatility, while simultaneously decreasing the fraction of 1° amines (by 28% points), leading to a lower required desorption temperature compared to TREN‐SY. Similarly, 0.54BO‐TREN‐SY showed a T_base‐des_ of 55°C with a WC_cyclic_ of 1.27 mmol g^−1^, which decreased to 1.18, 1.02, and 0.88 mmol g^−1^ at 50°C, 45°C, and 40°C, respectively (Figure 3c). These results clearly demonstrate that BO treatment can effectively lower the desorption temperature and enhance WC_cyclic_ by appropriately reducing the 1° amine content of TREN. Similar to the case of xBO‐PEI300‐SY, at 45°C the WC_cyclic_ of 0.54BO‐TREN‐SY surpassed that of 0.36BO‐TREN‐SY, again highlighting the balance between reduced CO_2_ uptake and enhanced desorption. For 0.72BO‐TREN‐SY, although the T_base‐des_ was reduced to 45°C, WC_cyclic_ dropped drastically to 0.32 mmol g^−1^ (Figure 3d). Notably, its WC_cyclic_ was even lower than that of 0.45BO‐PEI1200, which had fewer 1° amines. Thus, the severe decrease cannot be explained solely by the reduced 1° amine content. In the case of 0.72BO‐TREN‐SY, excessive BO loading substantially increased the proportion of 3° amines (from 25% to 37%) and caused the BO groups to concentrate near the molecular surface, thereby severely hindering CO_2_ access to the remaining 1° amines (Scheme S2).

Figure 4a summarizes the WC_cyclic_ values of all samples at various desorption temperatures. At 70°C, WC_cyclic_ was measured only for the Amine‐SY samples, where PEI300‐SY and PEI1200‐SY exhibited relatively high values of 1.58 and 1.41 mmol g^−1^, respectively. Between 55°C and 65°C, 0.15BO‐PEI300‐SY displayed the highest WC_cyclic_, followed by 0.36BO‐TREN‐SY. At 50°C, 0.30BO‐PEI300‐SY showed the highest WC_cyclic_, followed by 0.36BO‐TREN‐SY. At 45°C, 0.30BO‐PEI300‐SY and 0.54BO‐TREN‐SY exhibited the highest values in the same order. At 40°C, 0.54BO‐TREN‐SY showed the highest WC_cyclic_, slightly exceeding that of 0.30BO‐PEI300‐SY. Notably, the xBO‐PEI1200‐SY samples generally exhibited poorer WC_cyclic_ performance than both xBO‐PEI300‐SY and xBO‐TREN‐SY across most desorption temperatures. This can be attributed to their much higher proportion of 3° amines compared to xBO‐PEI300‐SY and their significantly lower proportion of 1° amines compared to xBO‐TREN‐SY. In contrast, the xBO‐PEI300‐SY series exhibited the best performance at all desorption temperatures except 40°C (Figure 4a). This behavior can be attributed to the molecular structure of PEI300, which possesses a very low fraction of tertiary amines (15%) compared to the other amines, resulting in a high proportion of highly CO_2_‐reactive 1° and 2° amines. At the same time, the moderate fraction of 1° amines (44%) compared to that of TREN (75%), enables facile CO_2_ desorption even at low desorption temperatures.

These results led to several key conclusions. First, among the three amines tested, PEI1200 exhibited the least effective response to BO functionalization. This is likely due to its inherently low 1° amine content and large molecular size, which limits CO_2_ accessibility to internal amine sites after BO treatment. This is further supported by MD simulations, which will be discussed in Section 2.5. Second, a low level of BO functionalization in PEI300 and TREN enabled high WC_cyclic_ values (1.42–1.60 mmol g^−1^) at desorption temperatures of 55°C–65°C. Third, moderate BO functionalization of these two amines resulted in decent WC_cyclic_ values (1.09–1.27 mmol g^−1^) at lower desorption temperatures of 45°C–50°C. Fourth, even at 40°C, moderate BO functionalization achieved reasonable WC_cyclic_ values (0.86–0.88 mmol g^−1^). Fifth, excessive BO treatment led to a significant decline in WC_cyclic_, highlighting the importance of optimizing the BO content. Finally, materials showing the highest WC_cyclic_ values at 40°C–65°C had their 1° amine contents in the range of 24%–33%. This suggests that to achieve high working capacities at low regeneration temperatures, it is advisable to use moderately BO‐functionalized PEI300 or TREN, targeting a 1° amine content within the 24%–33% range.

Our experimental screening showed that 0.30BO‐PEI300‐SY and 0.54BO‐TREN‐SY have the highest WC_cyclic_ values at low desorption temperatures of 45°C and 40°C, respectively. The optimal adsorbent was not the material with the highest initial CO_2_ uptake under low‐temperature desorption conditions. Instead, materials in which the amine‐CO_2_ interactions have been moderated by an appropriate degree of BO functionalization exhibited superior WC_cyclic_ values. In other words, by tuning the extent of BO functionalization, we can achieve a balance between reasonable WC_cyclic_ and energy requirements for regeneration.

We additionally performed a simplified calculation of the thermal energy required to produce a unit mass of CO_2_ (MJ kg_CO2_^−1^) in a temperature vacuum swing adsorption (TVSA) system (Figure 4b) [49, 50]. These calculations were performed for two optimal materials (0.30BO‐PEI300‐SY and 0.54BO‐TREN‐SY), along with several previously reported benchmark chemisorbents and physisorbents for DAC. For chemisorbents, three materials whose process‐level energy consumption has already been evaluated were selected: amine‐grafted cellulose hydrogel (APDES), silica gel (TPMS), and mesoporous silica powder (TMCM‐41) [50]. For physisorbents, NbOFFIVE‐1‐Ni (NbOFFIVE) and SIFSIX‐18‐Ni‐β (SIFSIX)–widely recognized as among the best‐performing materials in this category–were chosen for comparison. The calculated thermal energy consumptions of 0.30BO‐PEI300‐SY and 0.54BO‐TREN‐SY were 2.91 and 2.85 MJ kg_CO2_^−1^, respectively (Figure 4c). Although TMCM‐41 and APDES have been reported to exhibit relatively low energy consumption among the benchmark sorbents, their specific energy requirements are still 5.12 and 5.08 MJ kg_CO2_^−1^, respectively [50]. This can be attributed to the requirement for desorption temperatures exceeding 100°C, despite their relatively high working capacities of 0.95 and 1.1 mmol g^−1^. These comparisons clearly indicate that our materials require substantially lower energy input to capture comparable amounts of CO_2_, demonstrating their superior energy efficiency.

To investigate the effect of BO modification of amines on the CO_2_ adsorption kinetics, the CO_2_ uptake measured by TGA was analyzed using the Avrami equation (Figure S20). The calculated adsorption rate constant (k, min^−1^) for the xBO‐PEI1200‐SY series increased with increasing BO content, following the order: PEI1200‐SY (0.0082) < 0.15BO‐PEI1200‐SY (0.0121) < 0.30BO‐PEI1200‐SY (0.0123) < 0.45BO‐PEI1200‐SY (0.0161) (Table S2). A similar trend was also observed for the xBO‐PEI300‐SY and xBO‐TREN‐SY series. In other words, the adsorption rate increased with increasing BO incorporation, consistent with trends reported in previous studies [27, 51, 52, 53]. This behavior can be attributed to the increased spacing between amine groups introduced by BO functionalization, which provides additional free volume that facilitates rapid CO_2_ diffusion and enables easier access of CO_2_ molecules to the amine sites.

### 2.4 Evaluations Under More Realistic Conditions

**FIGURE 5:**
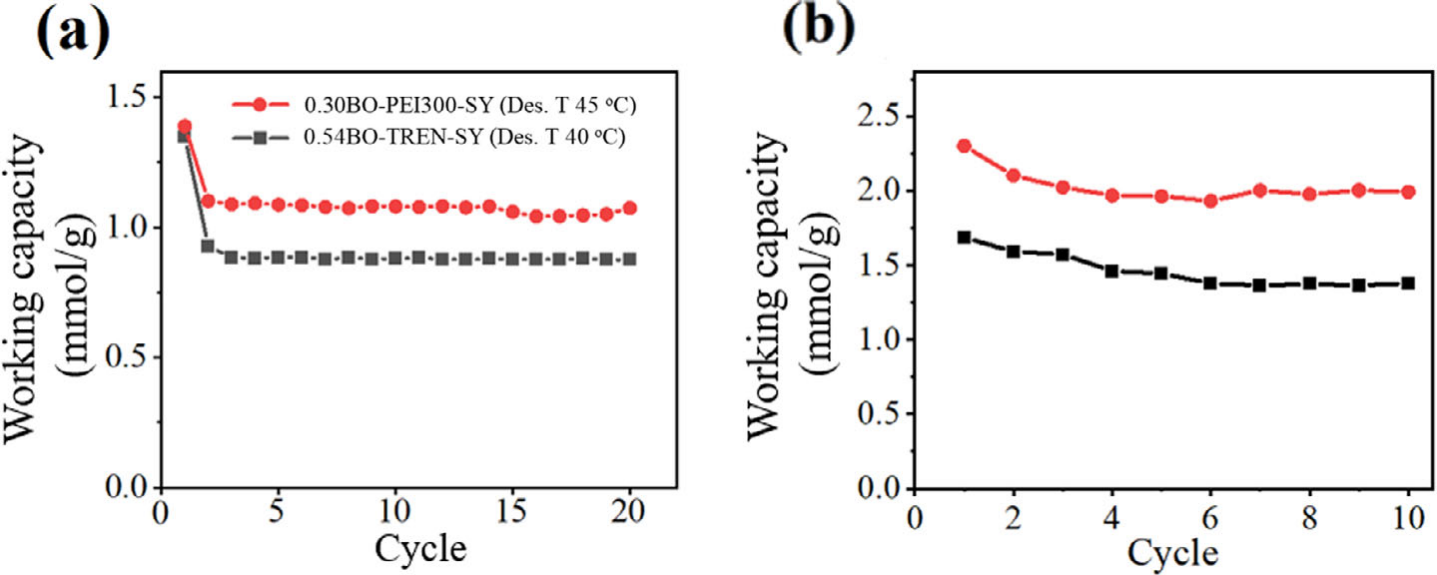
Working capacities of 0.30BO‐PEI300‐SY at a desorption temperature of 45°C and 0.54BO‐TREN‐SY at 40°C over (a) 20 consecutive adsorption–desorption TGA cycles under dry conditions (400 ppm CO_2_ in N_2_ balance) and (b) 10 consecutive adsorption–desorption breakthrough cycles under humid conditions (400 ppm CO_2_ in N_2_ balance with 50% RH).

In the previous section, two promising candidates (0.30BO‐PEI300‐SY and 0.54BO‐TREN‐SY) exhibited high CO_2_ working capacities at low desorption temperatures (40°C–45°C) during DAC sorbent screening using a gas mixture of 400 ppm CO_2_ and pure N_2_ balance. To further evaluate their DAC performance under more realistic conditions, 20 consecutive adsorption–desorption TGA cycles were conducted using air (79% N_2_, 21% O_2_) as the balance gas instead of pure N_2_ (Figure S21). As shown in Figure 5a, although both materials exhibited a large decrease in working capacity between the 1st and 2nd cycles, there were only slight reductions (2.8% for 0.30BO‐PEI300‐SY and 0.6% for 0.54BO‐TREN‐SY) over the subsequent 18 cycles. Notably, the results using air balance were almost identical to those measured using pure N_2_ balance (Figures 2c and 3c, and Figure S22), confirming that both sorbents maintained excellent stability even in the presence of oxygen. Additionally, a long‐term stability test over 100 cycles was conducted for 0.30BO‐PEI300‐SY, which exhibited the highest WC_cyclic_ value at low desorption temperatures (40°C–45°C). As shown in Figure S23, only negligible capacity loss was observed over 100 adsorption‐desorption cycles. Next, the working capacities of these two adsorbents were evaluated under 50% relative humidity (Figure S24). As shown in Figure 5b, both adsorbents exhibited higher CO_2_ uptakes in the 1st cycle compared to those obtained from TGA experiments under dry conditions, reaching 2.3 and 1.68 mmol g^−1^, respectively. Improved CO_2_ adsorption performance under humid conditions has been widely observed for many amine‐functionalized materials and is generally attributed to interactions among amines, H_2_O, and CO_2_ through various reaction mechanisms. The most well‐established pathways include stabilization of the zwitterionic intermediate by H_2_O followed by bicarbonate formation, promotion of carbamate ion‐pair or carbamic acid formation in the presence of H_2_O, and additional hydrogen‐bonding interactions involving amines induced by water molecules. Regardless of the specific pathway, water plays an auxiliary role in CO_2_ adsorption, ultimately leading to enhanced adsorption performance [53, 54, 55, 56, 57, 58, 59, 60, 61, 62, 63, 64].

Although both adsorbents showed a gradual decrease in working capacity during the initial cycles, their capacities stabilized around the 10th cycle, reaching 1.95 mmol g^−1^ for 0.30BO‐PEI300‐SY and 1.37 mmol g^−1^ for 0.54BO‐TREN‐SY, corresponding to decreases of 15.2% and 18.5% relative to the 1st cycle, respectively. These results demonstrate that both adsorbents can be stably employed as DAC sorbents even under realistic atmospheric conditions, i.e., in the presence of oxygen and moderate humidity.

To investigate the effect of BO functionalization on DAC performance under humid conditions, we also performed breakthrough experiments, with pristine TREN‐SY as a reference, up to the 3rd cycle under identical conditions. In the 1st cycle, TREN‐SY exhibited a higher working capacity (2.68 mmol g^−1^) than 0.54BO‐TREN‐SY. However, by the 3rd cycle, its working capacity decreased significantly to 1.02 mmol g^−1^ (Figure S25), falling below those of the BO‐functionalized materials. This represents a 62% reduction, which is greater than the capacity loss observed for the BO‐functionalized sorbent even after 10 cycles. These findings clearly demonstrate that BO functionalization significantly enhances the cyclic DAC performance of amine‐based sorbents under humid conditions.

Tables S5 and S6 compare the performances of 0.30BO‐PEI300‐SY and 0.54BO‐TREN‐SY with benchmark DAC sorbents in terms of WC_cyclic_ under dry and humid conditions. According to Table S5, under dry conditions, most previously reported materials exhibit WC_cyclic_ values in the range of 1.05–2.13 mmol g^−1^, with desorption temperatures between 80°C and 150°C. Notably, high‐performing sorbents such as 40TEPA‐10PEG/SiO_2_ and Na[TFPA]‐F‐COF show desorption temperatures around 90°C, while Cr‐MIL‐101‐SO_3_H‐TETA, which exhibits the lowest desorption temperature of 80°C, has a WC_cyclic_ value of 1.12 mmol g^−1^. In contrast, 0.30BO‐PEI300‐SY and 0.54BO‐TREN‐SY demonstrate competitive WC_cyclic_ values of approximately 1 mmol g^−1^ while achieving remarkably low desorption temperatures of 45°C and 40°C, respectively. Similarly, Table S6 presents sorbent performance under humid conditions, where WC_cyclic_ ranges from 0.85 to 3.36 mmol g^−1^. Among these, PEI@H‐SiO_2_, PEI/PME, and COF‐999 exhibit exceptionally high adsorption capacities, with the respective desorption temperatures of 110°C, 90°C, and 60°C. PEHA‐PO‐1‐2/50S, which exhibits a relatively low desorption temperature of 50°C, has a WC_cyclic_ value of 1.22 mmol g^−1^. In comparison, 0.30BO‐PEI300‐SY and 0.54BO‐TREN‐SY demonstrate outstanding performance under humid conditions as well, showing WC_cyclic_ values of 1.99 and 1.37 mmol g^−1^, respectively, at desorption temperatures of only 45°C and 40°C. These results clearly indicate that, compared with benchmark sorbents, our materials combine excellent WC_cyclic_ values with significantly lower desorption temperatures, making them highly energy‐efficient and promising candidates for practical DAC applications.

### 2.5 Mechanistic Analysis via MD Simulations

From the experimental observations in Sections 2.3 and 2.4, only the total amount of adsorbed CO_2_ could be determined for each sorbent, while the individual contributions of CO_2_ adsorbed on 1°, 2°, and 3° amines within each sorbent could not be distinguished. Therefore, MD simulations were performed to predict the amount of CO_2_ adsorbed on each type of amine (1°, 2°, and 3°) in both pristine and BO‐treated amine sorbents. To construct systems comparable to the experimental samples, two sets of models were developed: one containing only pristine amines and another representing xBO‐Amine systems with a similar ratio of introduced BO groups compared to the experimentally used amount. MD simulations were then carried out to analyze the CO_2_ adsorption behavior under a nitrogen atmosphere.

**FIGURE 6:**
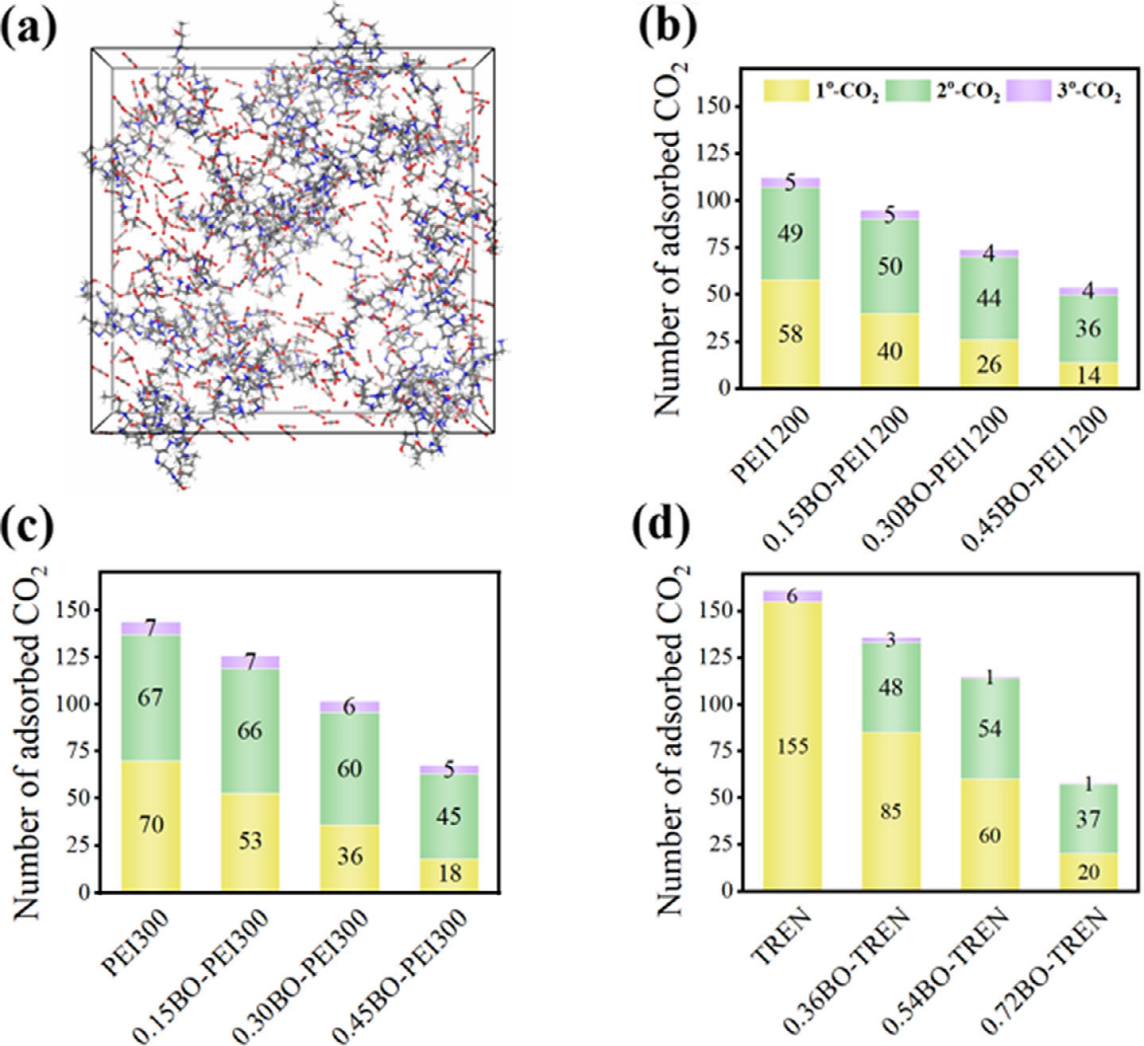
(a) Snapshots of CO_2_ adsorption on 0.30BO‐PEI300 as a representative structure (N_2_ are omitted for clarity), and the number of CO_2_ molecules adsorbed on 1°, 2°, and 3° amines for (b) xBO‐PEI1200, (c) xBO‐PEI300, and (d) xBO‐TREN obtained from MD simulations.

The overall trends in the simulation results are consistent with those observed experimentally in a single adsorption cycle (Figure 6; Figure S26–S28 and Table S4). For pristine amines, the total CO_2_ adsorption amount from simulations followed the order TREN (161) > PEI300 (144) > PEI1200 (112), in good agreement with the 1st–2nd cycle TGA results shown in Figures 1a, 2a, and 3a. A closer examination of the MD results revealed that this order corresponded well to the amount of CO_2_ adsorbed on 1° amines, namely TREN (155) > PEI300 (70) > PEI1200 (58). In contrast, the adsorption on 2° and 3° amines showed no clear correlation with the total CO_2_ adsorption amount. This indicates that CO_2_ adsorption on 1° amine sites determines the overall CO_2_ capture capacity of pristine amines.

To further clarify why the CO_2_ adsorption on 1° amines followed the order TREN > PEI300> PEI1200, the CO_2_ uptake per 1° amine group was calculated and found to follow the sequence TREN (2.07) > PEI300 (1.77) > PEI1200 (1.57). This can be attributed to the reduced accessibility of CO_2_ molecules to the internal 1° amine sites as the molecular weight of the amine increases, since longer molecular chains tend to become more easily entangled.

Moreover, for all amines, the total CO_2_ adsorption amount decreased dramatically with BO treatment, consistent with the experimental observations. Specifically, while CO_2_ adsorption on 2° and 3° amines changed little after BO modification, there was a substantial decrease for 1° amines. This confirms that the reduction in CO_2_ uptake upon BO treatment primarily originates from the loss of accessible 1° amine sites, due to (1) the conversion of 1° amines into 2° or 3° amines via covalent bonding with BO groups and (2) the reduced accessibility caused by steric hindrance from the BO groups. These findings strongly support our hypothesis that the pronounced decrease in CO_2_ adsorption on 1° amines is the key factor responsible for the reduced adsorption capacity and lower desorption temperature observed after BO treatment.

In the case of 2° amines, CO_2_ adsorption either increased or decreased after BO treatment depending on the specific system, which can be ascribed to the combined effects of a larger proportion of 2° amines and the steric hindrance induced by neighboring BO groups. An increase in CO_2_ adsorption on 2° amines was only observed in two cases: PEI1200 → 0.15BO‐PEI1200 and 0.36BO‐TREN → 0.54BO‐TREN. In all other cases, CO_2_ adsorption on 2° amines decreased after BO treatment, likely due to the dominant steric hindrance effect caused by the adjacent BO groups, which reduces the exposure of 2° amines by repositioning them within more compact or internally buried regions of the polymer matrix. Additionally, a larger number of BO groups may promote intra‐ or intermolecular hydrogen bonding, further restricting chain mobility and enhancing local structural rigidity [65]. Such conformational constraints can modulate the accessibility of 2° amines in a system‐dependent manner, ultimately suppressing their interaction with CO_2_. Interestingly, although the fraction of 3° amines also increased with BO treatment, the overall adsorption capacity did not change significantly. This observation is consistent with previous reports that while 3° amines may form bicarbonate species with the aid of H_2_O under humid conditions, they exhibit very limited reactivity with CO_2_ under dry conditions [66, 67].

## 3 Conclusions

This study aimed to develop a DAC sorbent capable of achieving a high WC_cyclic_ at low desorption temperatures (<70°C) by optimizing both the type of amine impregnated into the mesoporous silica support and the degree of epoxide functionalization. BO functionalization was applied to three representative amines‐(PEI1200, PEI300, and TREN)‐ with varying molecular weights and amine compositions, enabling regulation of the CO_2_ adsorption strength by tuning the fractions of 1°, 2°, 3° amines.

To investigate the effect of BO functionalization on the WC_cyclic_ and desorption temperature, TGA experiments were conducted over five adsorption–desorption cycles at varied desorption temperatures. Upon reducing the desorption temperature, materials rich in 1° amines suffered a sharp decline in WC_cyclic_ due to a sharp increase in undesorbed CO_2_, while BO‐treated materials with moderate 1° amines exhibited higher WC_cyclic_ owing to lower desorption energy requirements. Through systematic screening, 0.30BO‐PEI300‐SY and 0.54BO‐TREN‐SY were identified as the optimal sorbents, exhibiting the highest WC_cyclic_ at low desorption temperatures of 45°C and 40°C, respectively. Remarkably, both materials maintained excellent WC_cyclic_ values at substantially lower desorption temperatures than most reported DAC sorbents. This finding suggests that moderately BO‐functionalized PEI300 or TREN, with 1° amine fractions in the range of 24%–33%, provides an effective strategy for achieving high WC_cyclic_ at low regeneration temperatures. Under oxygen‐rich and moderately humid conditions, both optimal sorbents also exhibited excellent stability. Notably, BO functionalization significantly enhanced the cyclic DAC performance of the amine‐based sorbents under humid conditions, enabling 0.30BO‐PEI300‐SY and 0.54BO‐TREN‐SY to achieve high WC_cyclic_ values of 1.99 and 1.37 mmol g^−1^, respectively, even at low desorption temperatures of 45°C and 40°C. These results clearly demonstrate the sorbents’ superior energy efficiency and excellent potential for practical DAC applications.

Furthermore, MD simulations revealed that CO_2_ adsorption on 1° amine sites plays a dominant role in determining the overall CO_2_ capture capacity of pristine amines. The simulation results also confirmed that the observed reduction in CO_2_ uptake after BO treatment primarily originates from the loss of accessible 1° amine sites, both through their conversion by BO and the reduced accessibility caused by steric hindrance from neighboring BO groups. These findings strongly support the hypothesis that the pronounced decrease in CO_2_ adsorption on 1° amines is the key factor responsible for both the lower CO_2_ capture capacity and reduced desorption temperature observed after BO functionalization.

## 4 Experimental Section

### 4.1 Materials

Sylysia 350 (SY350) was purchased from Fuji‐Silysia. Butylene oxide (BO, 72.11 g mol^−1^) was obtained from Sigma–Aldrich. Branched PEI with average molecular weights of 1200 (PEI1200) and 300 (PEI300), as well as tris(2‐aminoethyl)amine (TREN, 98%), were purchased from Thermo Fisher Scientific.

### 4.2 Syntheses of xBO‐Amine and xBO‐Amine‐SY

BO functionalization of amines was performed using a previously reported method with slight modifications [27]. Specifically, 0.3 g of each amine was dissolved in methanol to prepare a 20 wt.% solution. A desired amount of BO was then added dropwise to the amine solution, followed by vigorous stirring at room temperature for 12 h. The resulting BO‐functionalized amines were denoted as xBO‐Amine, where x represents the initial molar ratio of BO to nitrogen atoms (O/N ratio) in the amine (PEI1200, PEI300, or TREN). Subsequently, 0.3 g of SY350 was added to each xBO‐Amine solution, followed by wet impregnation using a rotary evaporator under vacuum. The resulting materials were dried in a vacuum oven at 60°C for 12 h. The BO‐functionalized amines impregnated into SY350 were denoted as xBO‐Amine‐SY, where SY refers to SY350. The overall synthetic procedure is illustrated in Scheme 1.

### 4.3 Cyclic CO_2_ Adsorption–Desorption Experiments

A TGA 8000 (PerkinElmer) instrument was used to obtain CO_2_ adsorption–desorption profiles. Before the experiment, each sample was degassed at 100°C for 20 min under a nitrogen flow (40 mL/min) to remove any residual contaminants. Adsorption was carried out at 25°C using a gas mixture containing 400 ppm CO_2_ and N_2_ balance at a flow rate of 100 mL min^−1^. Desorption (regeneration) was performed using high‐purity N_2_ gas (99.9%) at 80 mL min^−1^, with regeneration temperatures ranging from 40°C to 70°C depending on the adsorbent. For each sample, CO_2_ adsorption–desorption profiles were obtained for 5 consecutive cycles. Based on previously reported protocols [28, 68, 69], the adsorption and desorption durations were set to 180 and 90 min, respectively.

To evaluate the oxidative stability of the two selected adsorbents, CO_2_ adsorption was performed at 25°C using the same gas flow conditions as described above, except that air (79% N_2_ and 21% O_2_) was used as the balance gas instead of N_2_. Desorption was conducted at 40°C–45°C using the same N_2_ flow conditions as described above. For each sample, CO_2_ adsorption–desorption profiles were recorded over 20 consecutive cycles, using the same adsorption and desorption durations as above. For 0.30BO‐PEI300‐SY, 100 repeated adsorption‐desorption cycles were additionally conducted under the same conditions mentioned above.

### 4.4 Characterization and Measurement

Detailed descriptions of the characterization, breakthrough experiments [39, 40, 41, 42], and MD simulations are provided in the Supporting Information.

## Author Contributions

J.Y. Han and Y.‐S. Bae conceptualized and initialized the research. J.Y. Han, Y. Ko, Y. Cho, S. Ravi, K.‐M. Ryoum, H.H. Han, Y. Choi, and C. Shin conducted the experiments under the guidance of Y.‐S. Bae. H. Jeong performed computational works under the guidance of J.W. Han. J.Y. Han and H. Jeong wrote the original draft. J.Y. Han and Y.‐S. Bae reviewed and revised the original draft.

## Conflicts of Interest

The authors declare no conflicts of interest.

## Supporting information




**Supporting file**: advs75091‐sup‐0001‐SuppMat.pdf

## Data Availability

The data that support the findings of this study are available from the corresponding author upon reasonable request.
