# Supplementary material for: Role of Epoxide Functionalization of Amines for Development of Direct Air Capture Sorbents with High Cyclic Working Capacity at Low Desorption Temperatures
Source: Adv Sci (Weinh). 2026 Apr 2;13(34):e75091. doi: 10.1002/advs.75091 (PMC13285134; doi:10.1002/advs.75091)
Supplement: Supplementary file 1 — Supporting file: advs75091‐sup‐0001‐SuppMat.pdf [file ADVS-13-e75091-s001.pdf]

Supporting Information

Role of Epoxide Functionalization of Amines for Development of Direct Air Capture Sorbents with High Cyclic Working Capacity at Low Desorption Temperatures

Joo Yeon Han[§], Hayoung Jeong[§], Younghyu Ko, Yohan Cho, Seenu Ravi, Kyu-Min Ryoum, Hyug Hee Han, Yujin Choi, Chaewon Shin, Jeong Woo Han, and Youn-Sang Bae**

J.Y. Han, Y. Ko, Y. Cho, S. Ravi, K.-M. Ryoum, H.H. Han, Y. Choi, C. Shin, and Prof. Y.-S. Bae

Department of Chemical and Biomolecular Engineering, Yonsei University, 50 Yonsei-ro, Seodaemun-gu, Seoul, 03722, Korea

E-mail: mowbae@yonsei.ac.kr

H. Jeong and J.W. Han

Department of Materials Science and Engineering, Research Institute of Advanced Materials, Seoul National University, 1 Gwanak-ro, Gwanak-gu, Seoul, 08826, Korea

E-mail: jwhan98@snu.ac.kr

[§]These authors contributed equally to this work.

Experimental Section

Characterization: Liquid-phase ^{13}C NMR spectra were measured at a ^{13}C frequency of 100.6 MHz using a 400 MHz FT-NMR spectrometer (Avance III HD 400). Amines with and without BO functionalization were dissolved in $CDCl_3$, and the spectra were recorded with an acquisition time of 1.36 s over 1024 transients. Inverse-gated proton decoupling was employed to suppress the Overhauser effect and enable quantitative analysis. All chemical shifts are reported in ppm relative to the solvent signal.
Nitrogen adsorption–desorption isotherms were measured at –196 °C using a 3Flex gas adsorption analyzer (Micromeritics). The sample was degassed under vacuum at 90 °C for 2 h to remove any contaminants before measurement. The BET method was used to determine the surface area of samples in a P/P_0 range of 0.05–0.30, and the total pore volume was calculated at $P/P_0 = 0.99$. The Barrett-Joyner-Halenda (BJH) method was used to determine the pore size distribution of each sample.

Cyclic Breakthrough Experiments Under Humid Conditions: Dynamic breakthrough experiments were conducted using a custom-built, fixed-bed system. Two mass flow controllers (0–100 mL min^{-1}, Bronkhorst) were used for a gas mixture containing 400 ppm CO_2 and N_2 balance. The first one produces a dry CO_2/N_2 stream, and the second one proceeds through a humidifier to generate a humid CO_2/N_2 stream saturated with water. A 400 ppm CO_2/N_2 stream with 50% relative humidity was prepared by combining the aforementioned dry and humid streams. A third mass flow controller (0–100 mL min^{-1}, Bronkhorst) directed a helium stream for the *in-situ* degassing and regeneration of the adsorbent packed in the column. The outlet flow rate was monitored using a 100-mL manual bubble flow meter (Supelco). The resulting humidity level and CO_2 concentration of the gas flow at the column outlet were measured online using a TES 1370 NDIR CO_2 Meter (TES Electrical Electronic Corp.). The as-synthesized powder samples were pelletized into binderless pellets with a size of 500–850 μm using a Carver press (Carver, Inc.). The obtained pellets were initially degassed at 90 °C for 2 h under vacuum and then packed into a stainless-steel column (15 cm × 0.44 cm). The remainder of the column was filled with glass beads and wool with a diameter of 750 μm. Prior to the experiment, the column was degassed under a helium flow (100 mL min^{-1}) at 100 °C for approximately 20 min to remove any adsorbed impurities during the packing procedure. Adsorption was carried out at 30 °C using a humid gas flow (100 mL min^{-1}) containing 400 ppm CO_2 and N_2 balance with 50% relative humidity, while

desorption was performed using 99.9% He gas (100 mL min^{-1}) at 40 or 45 °C. For each sample, adsorption and desorption breakthrough curves were obtained for 10 consecutive cycles of 180-min adsorption and 30-min desorption (**Scheme S1**).

MD Simulations: All-atom MD simulations were conducted using the Desmond simulation package (Schrödinger, Inc.) to investigate the structural behaviors of PEI and TREN, as well as their interactions with CO_2 molecules under a nitrogen atmosphere. The OPLS4 force field was employed to model all interatomic interactions, including amines, CO_2, and N_2 molecules. Initial configurations were constructed using the Disordered System Builder by randomly packing polymer chains into a cubic simulation box with periodic boundary conditions applied in all directions, thereby mimicking a dense and entangled polymer environment. All systems contained 50 polymer chains of PEI1200, PEI300, TREN, or their BO derivatives. To reflect structural differences among the amine-functionalized polymers, the initial ratios of 1°:2°:3° amines were set to be close to those reported for PEI1200, PEI300, and TREN. To model the BO-modified amines, BO units were introduced onto the polymer backbones based on these initial amine distributions, resulting in partial conversion of 1° amines to 2° or 3° amines. Here, 1°: 2°: 3° ratios closely matched the compositions of experimental samples. To simulate adsorption behavior under realistic mixed-gas conditions, 400 CO_2 and 400 N_2 molecules were inserted into each simulation box prior to equilibration. All gas molecules were initially distributed uniformly and randomly throughout the simulation box. This approach was adopted to both accommodate limitations in directional gas implementation and more accurately reflect an equilibrium-like distribution within the polymer matrix. Under these conditions, the simulation focuses on the spatial accessibility of amine groups and their interactions with nearby CO_2 molecules, rather than diffusion kinetics. To minimize the effects of initial configuration bias, 10 independent structural models were generated for each system. Structures exhibiting unphysical features, such as excessive polymer chain overlapping or clustered gas molecules, were excluded from further analysis. All remaining models underwent equilibration under NPT conditions to allow volume relaxation and molecular rearrangement, ensuring that subsequent analyses reflected thermodynamically stable configurations. Representative structures were selected based on consistent behavior observed across replicates, supported by radial distribution function (RDF) analyses of CO_2-amine interactions. To ensure proper system relaxation and eliminate steric hindrance within the densely packed polymer matrix, a stepwise equilibration protocol

was employed prior to production runs. The procedure began with two short Brownian dynamics simulations: one under the NVT ensemble at 10 K with a 1 fs timestep and another under anisotropic NPT conditions at 100 K and 1.01325 bar using a 2 fs timestep. These steps were designed to gradually stabilize the system while preserving structural integrity. Subsequently, the system was equilibrated through MD under the NPT ensemble at 300 K and 1.01325 bar for 0.1 ns, followed by additional equilibration steps consisting of a 0.5 ns NVT simulation and a 0.5 ns NPT simulation, both at 298 K and using a 2 fs timestep. These stages were implemented to maintain thermal and volumetric stability prior to data collection. To examine the CO_2 adsorption process, production runs were executed under the NVT ensemble. The adsorption stage was simulated for 5.0 ns at 298 K. Each production run employed a 2 fs timestep, and trajectory snapshots were saved every 100 fs for post-simulation analysis.

Trajectory analyses were performed to quantify CO_2 adsorption behavior and structural features of the systems. Adsorbed CO_2 molecules were identified using a distance cutoff of 4 Å from any amine nitrogen atom. Each CO_2 molecule was then assigned to its nearest nitrogen atom, which was classified as 1°, 2°, or 3° according to its local bonding geometry. For each pristine Amine and xBO-Amine system, the relative CO_2 adsorption amounts on 1°, 2°, and 3° amines under a nitrogen atmosphere were calculated using the procedure described above.

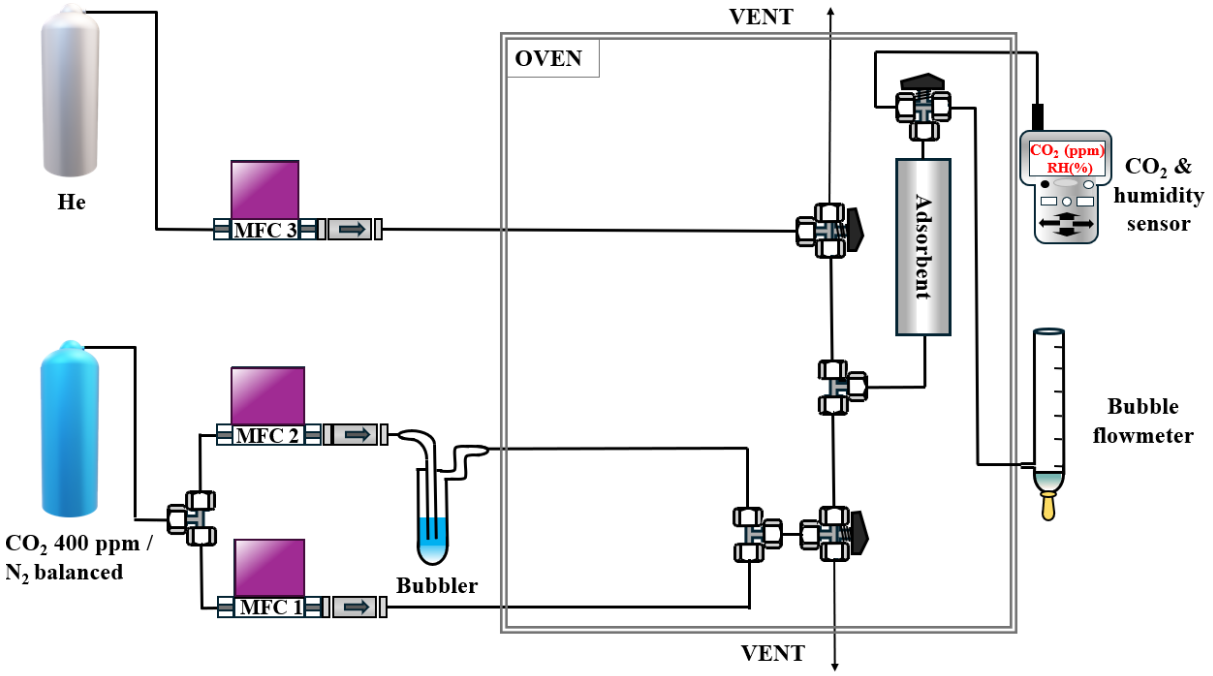

Scheme S1. Schematic of the dynamic breakthrough experimental setup.

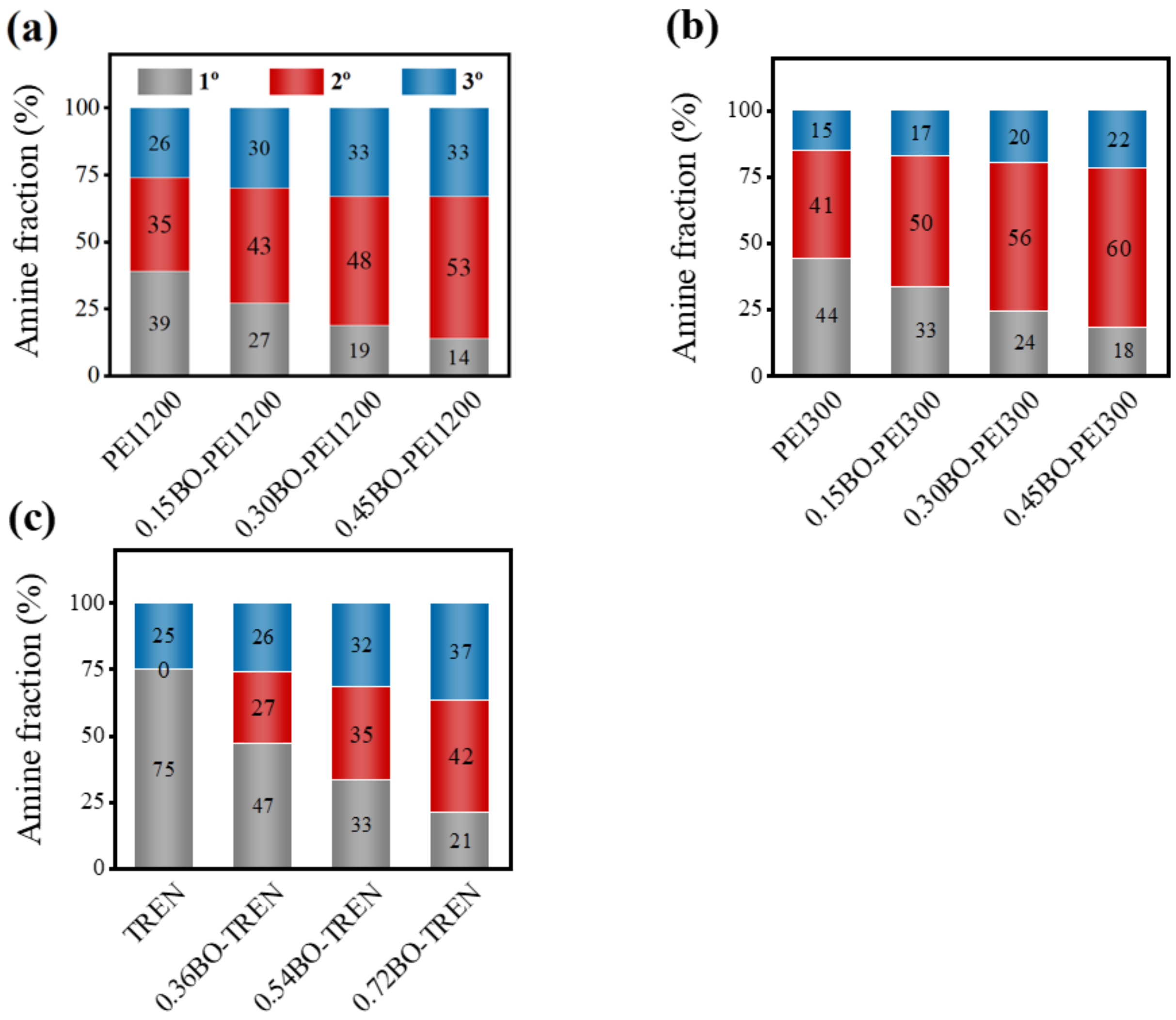

Figure S1. 1°:2°:3° amine ratios of (a) xBO-PEI1200, (b) xBO-PEI300, and (c) xBO-TREN.

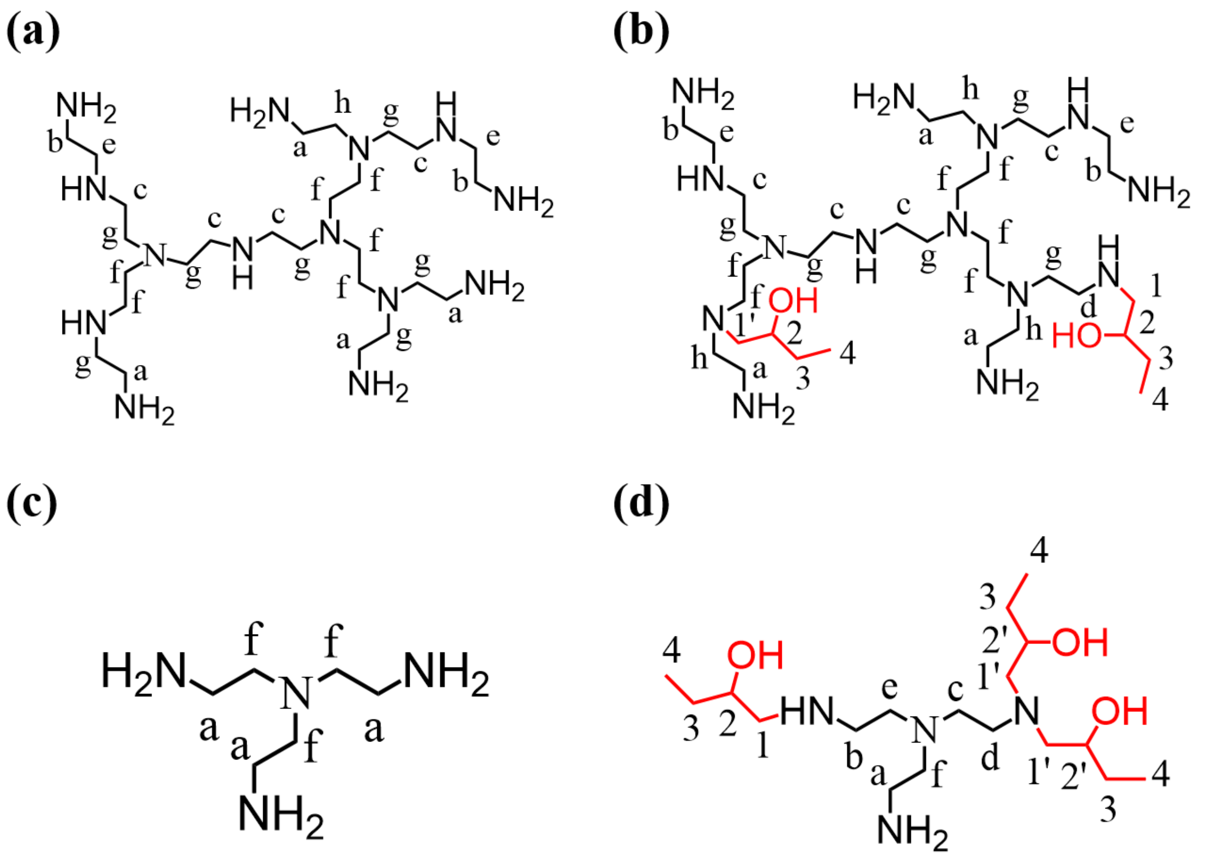

Figure S2. Molecular structures of (a) PEI, (b) BO-PEI, (c) TREN, and (d) BO-TREN. The letters and numbers indicate the ^{13}C NMR peaks.

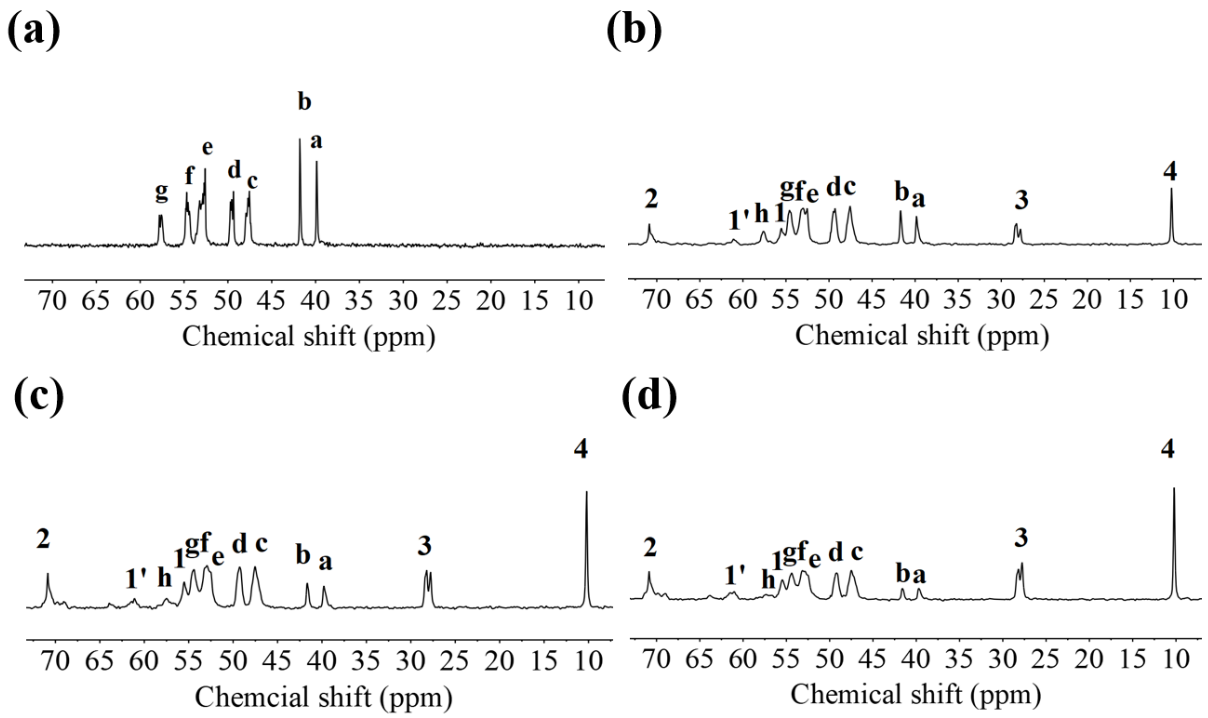

Figure S3. ^{13}C NMR spectra of (a) PEI1200, (b) 0.15BO-PEI1200, (c) 0.30BO-PEI1200, and (d) 0.45BO-PEI1200. The 1°:2°:3° amine ratios were calculated as ($A_a + A_b$): ($A_c + A_d + A_e + A_1$)/2: ($A_f + A_g + A_h + A_{1'}$)/3. A_i denotes the integrated peak area corresponding to species i.

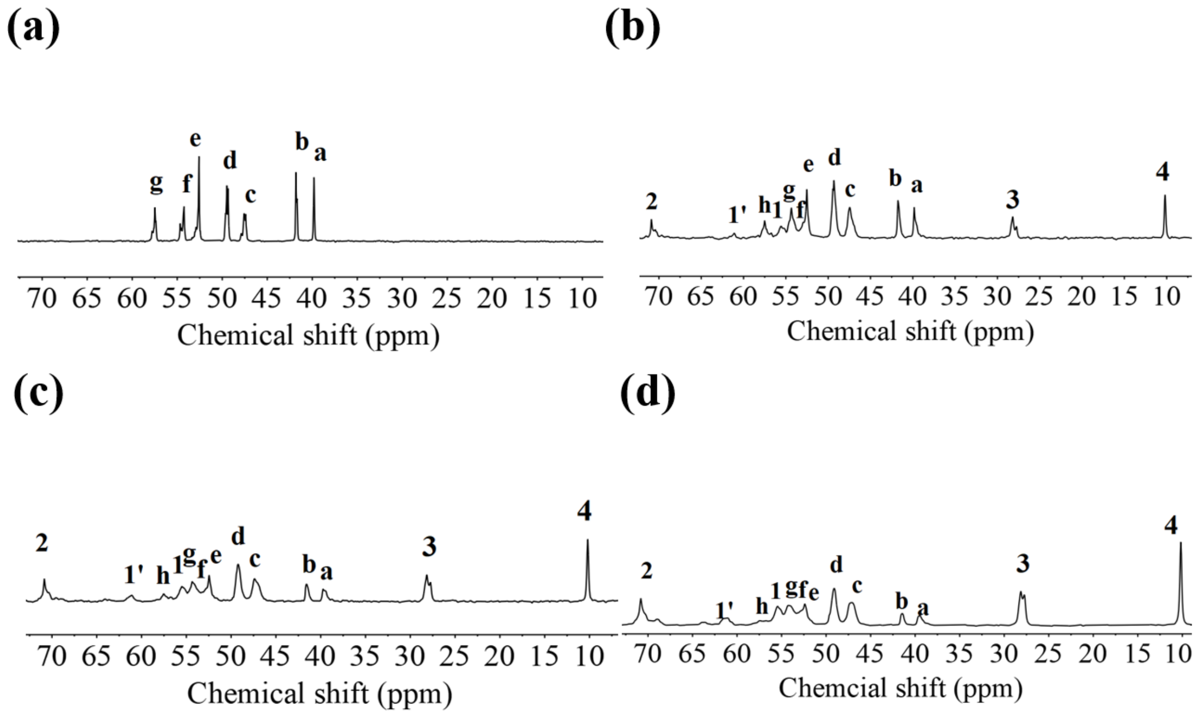

Figure S4. ^{13}C NMR spectra of (a) PEI300, (b) 0.15BO-PEI300, (c) 0.30BO-PEI300, and (d) 0.45BO-1200PEI300. The 1°:2°:3° amine ratios were calculated as $(A_a + A_b)$: $(A_c + A_d + A_e + A_1)/2$: $(A_f + A_g + A_h + A_{1'})/3$. A_i denotes the integrated peak area corresponding to species i.

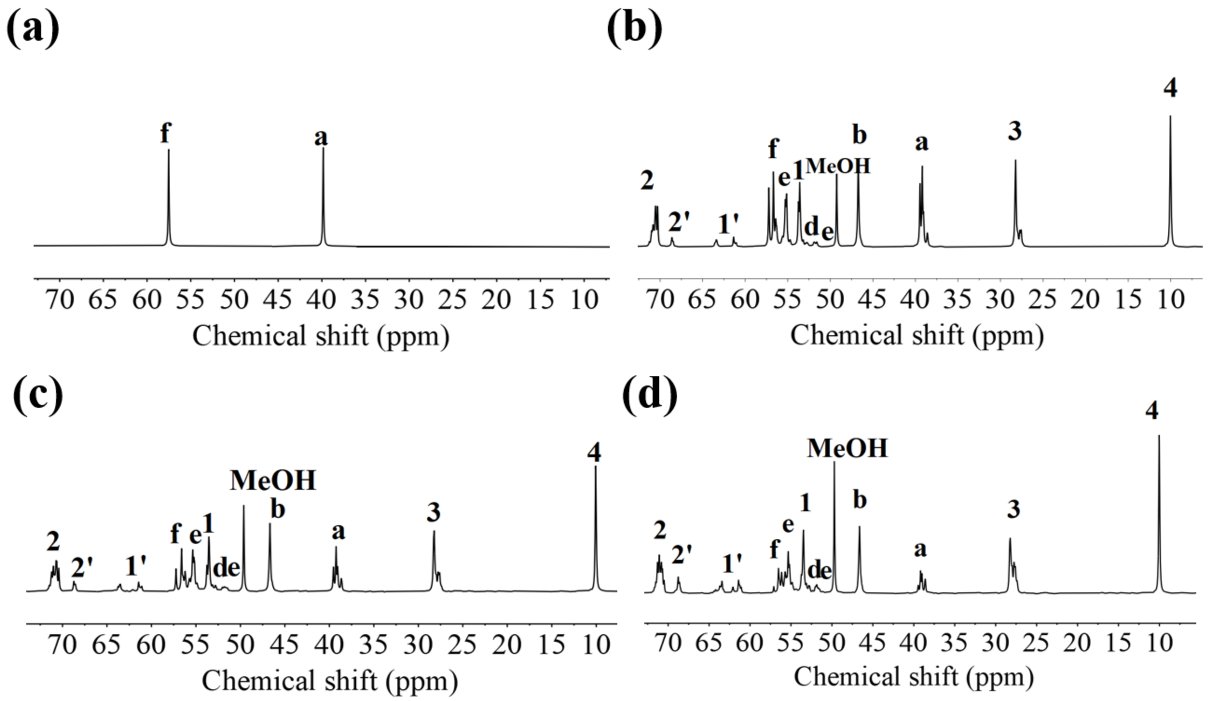

Figure S5. ^{13}C NMR spectra of (a) TREN, (b) 0.36BO-TREN, (c) 0.54BO-TREN, and (d) 0.72BO-TREN. The 1°:2°:3° amine ratios were calculated as (A_a): (A_b + A_1)/2: (A_c + A_d + A_e + A_f + $A_{1'}$)/3. A_i denotes the integrated peak area corresponding to species i.

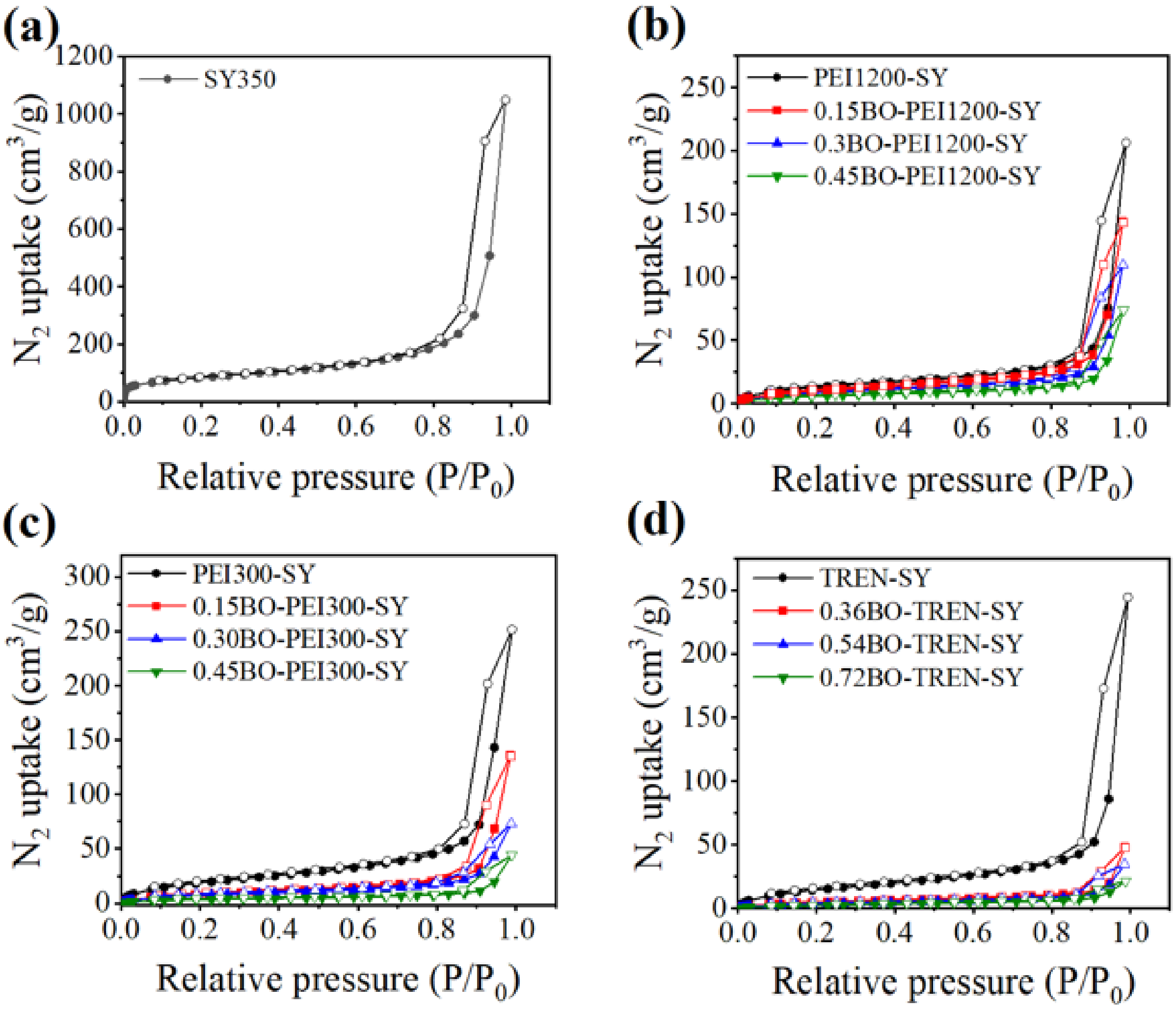

Figure S6. N_2 adsorption–desorption isotherms of (a) SY350 (SY), (b) PEI1200-SY and xBO-PEI1200-SY, (c) PEI300-SY and xBO-PEI300-SY, and (d) TREN-SY and xBO-TREN-SY.

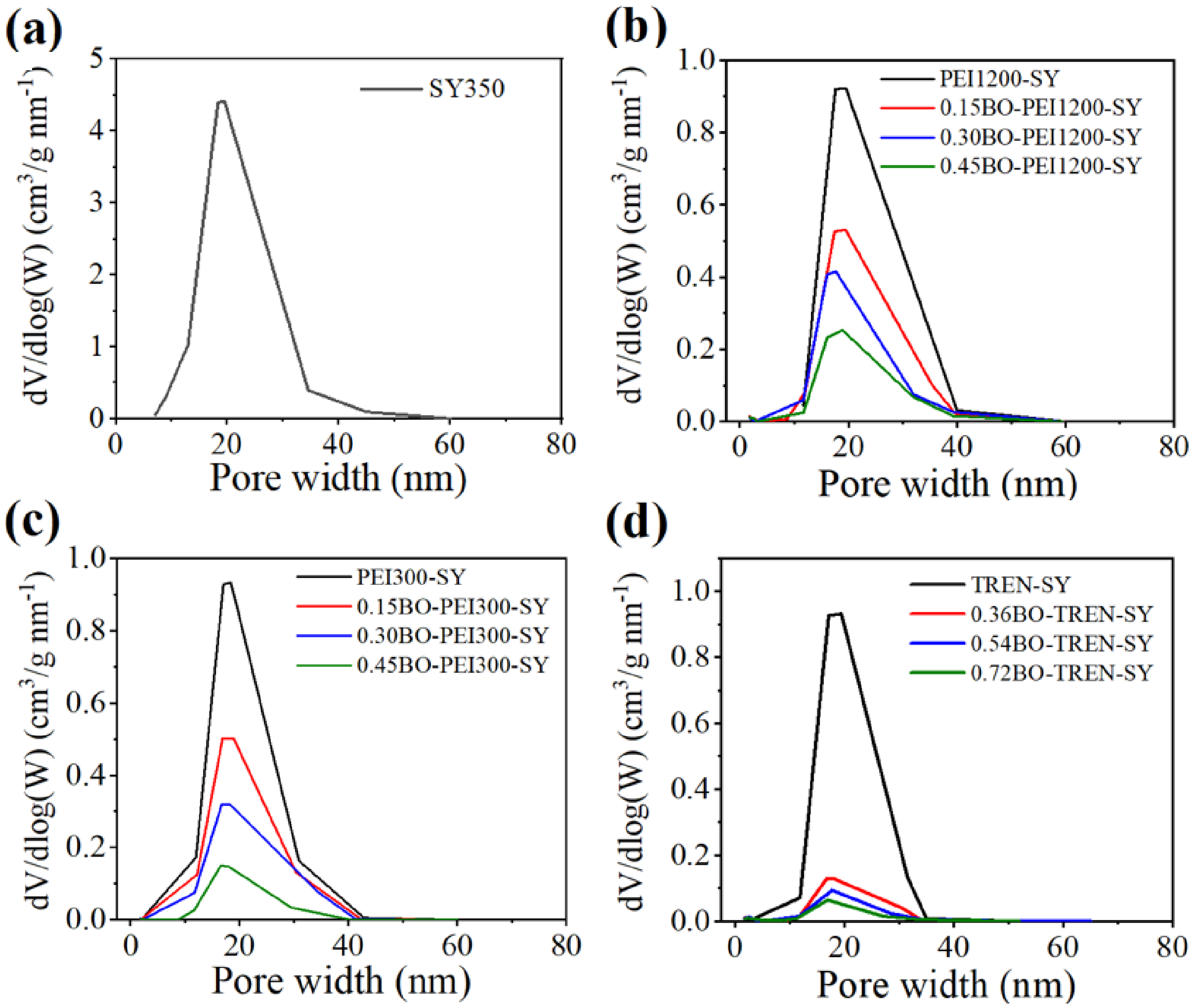

Figure S7. Pore size distributions of (a) SY350, (b) PEI1200-SY and xBO-PEI1200-SY, (c) PEI300-SY and xBO-PEI300-SY, and (d) TREN-SY and xBO-TREN-SY.

Table S1. BET surface areas (S_{BET}) and total pore volumes (V_{total}) of Amine-SY and xBO-Amine-SY materials.

Sample	S_{BET} ($m^2\ g^{-1}$)	V_{total} ($cm^3\ g^{-1}$)	Sample	S_{BET} ($m^2\ g^{-1}$)	V_{total} ($cm^3\ g^{-1}$)	Sample	S_{BET} ($m^2\ g^{-1}$)	V_{total} ($cm^3\ g^{-1}$)
PEI1200	58.4	0.38	PEI300	55.8	0.37	TREN	54.3	0.35
0.15BO-PEI1200	40.8	0.22	0.15BO-PEI300	33	0.21	0.36BO-TREN	18.6	0.08
0.30BO-PEI1200	33.3	0.17	0.30BO-PEI300	29.1	0.17	0.54BO-TREN	14.4	0.05
0.45BO-PEI1200	23	0.11	0.45BO-PEI300	13.3	0.06	0.72BO-TREN	10.2	0.03

S_{BET} and V_{total} of SY were 294.8 $m^2\ g^{-1}$ and 1.63 $cm^3\ g^{-1}$, respectively.

For clarity, -SY has been omitted from the sample names.

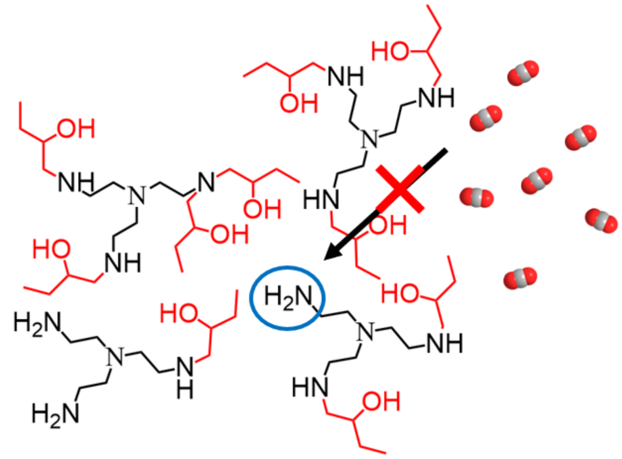

Scheme S2. Schematic of reduced CO_2 accessibility to primary amines in excessively BO-functionalized TREN.

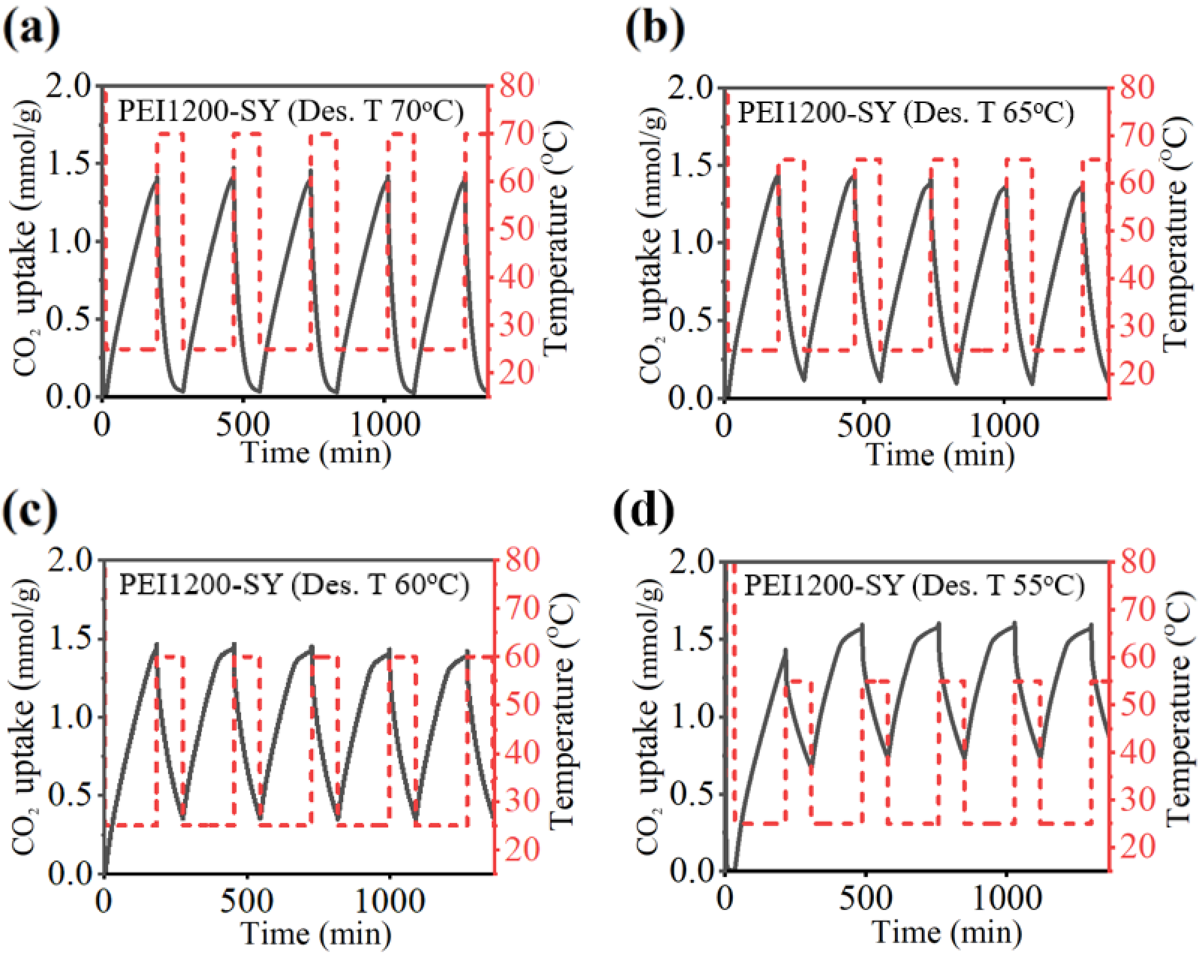

Figure S8. TGA adsorption–desorption curves of PEI1200-SY measured at desorption temperatures of (a) 70 °C, (b) 65 °C, (c) 60 °C, and (d) 55 °C.

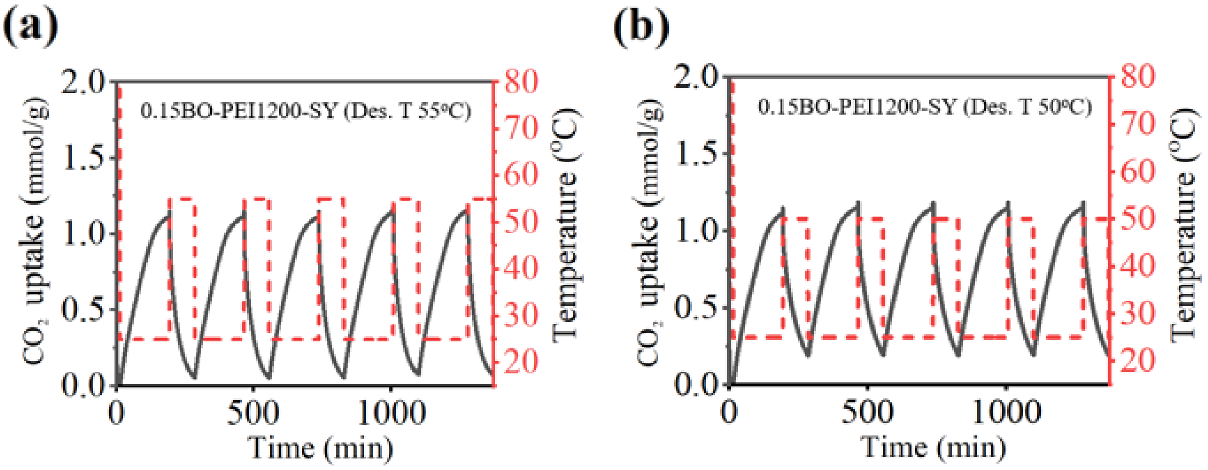

Figure S9. TGA adsorption–desorption curves of 0.15BO-PEI1200-SY measured at desorption temperatures of (a) 55 °C and (b) 50 °C.

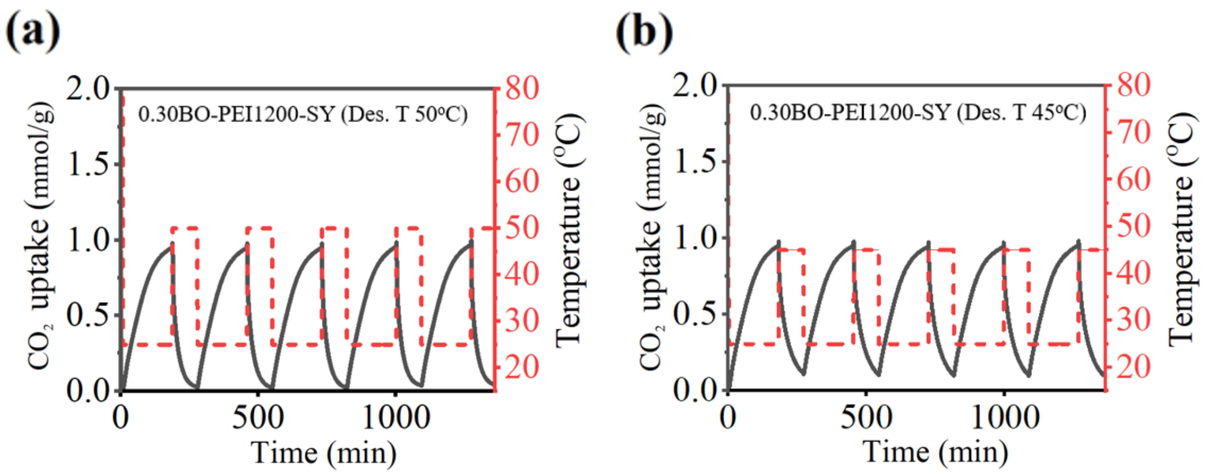

Figure S10. TGA adsorption–desorption curves of 0.30BO-PEI1200-SY measured at desorption temperatures of (a) 50 °C and (b) 45 °C.

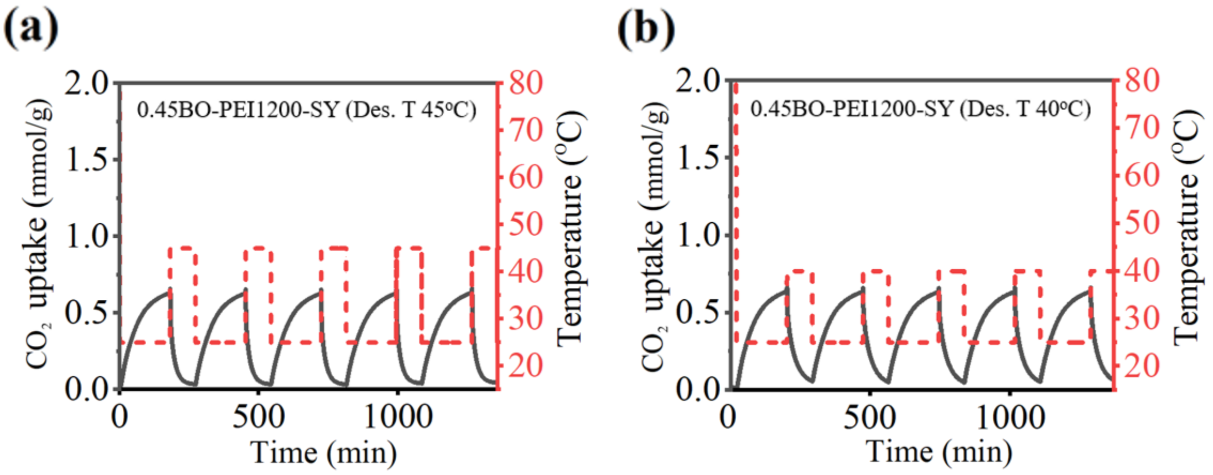

Figure S11. TGA adsorption–desorption curves of 0.45BO-PEI1200-SY measured at desorption temperatures of (a) 45 °C and (b) 40 °C.

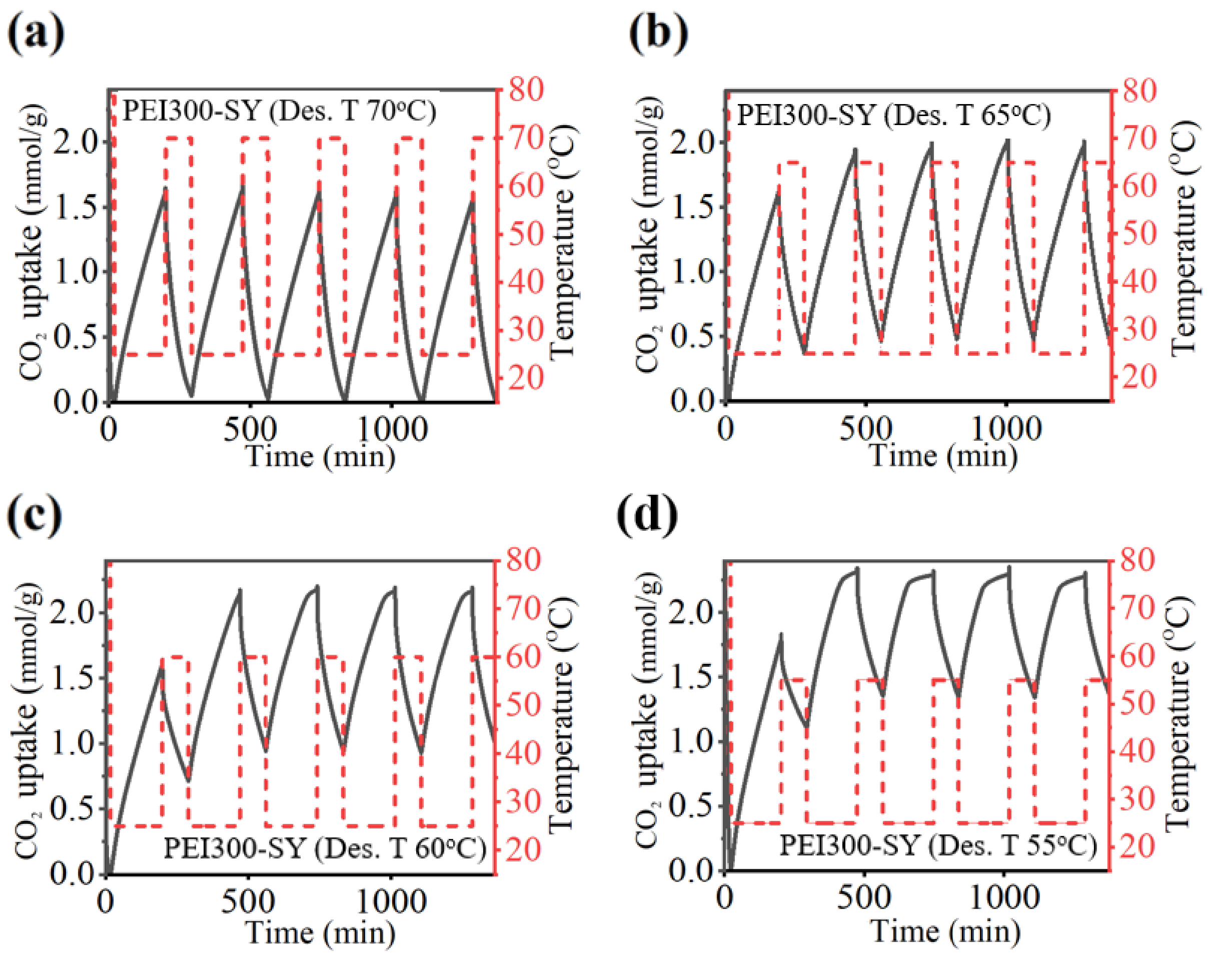

Figure S12. TGA adsorption–desorption curves of PEI300-SY measured at desorption temperatures of (a) 70 °C, (b) 65 °C, (c) 60 °C, and (d) 55 °C.

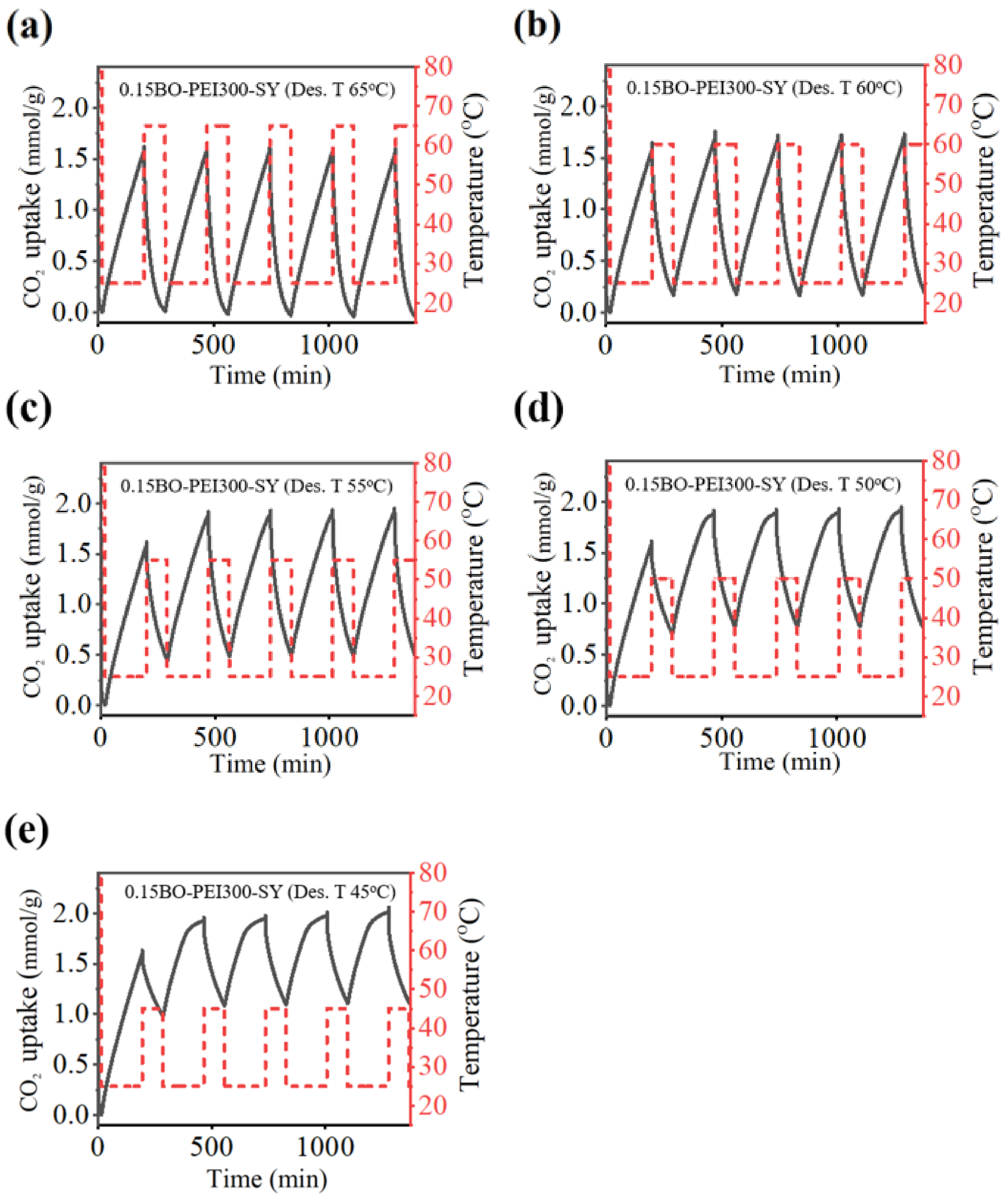

Figure S13. TGA adsorption–desorption curves of 0.15BO-PEI300-SY measured at desorption temperatures of (a) 65 °C, (b) 60 °C, (c) 55 °C, (d) 50 °C, and (e) 45 °C.

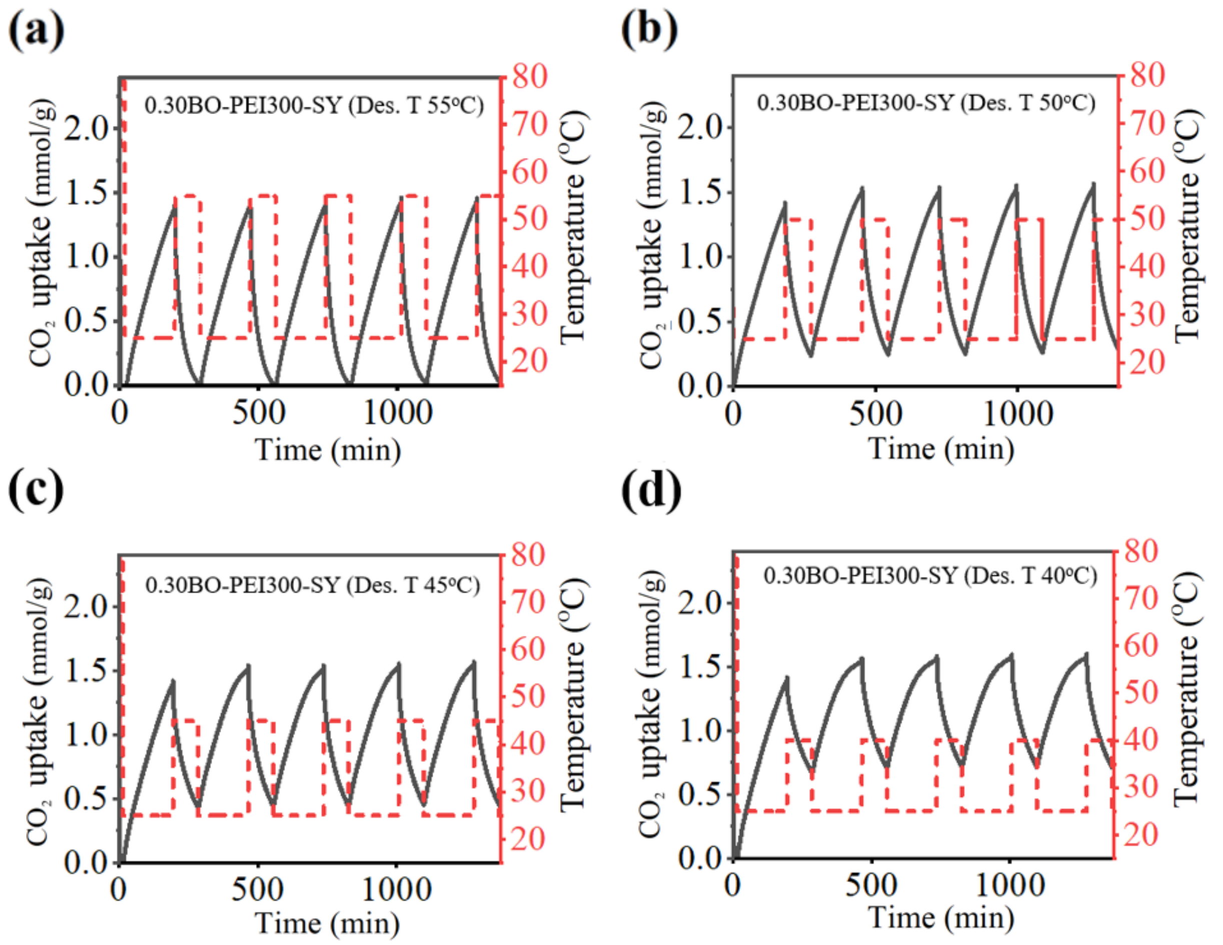

Figure S14. TGA adsorption–desorption curves of 0.30BO-PEI300-SY measured at desorption temperatures of (a) 55 °C, (b) 50 °C, (c) 45 °C, and (d) 40 °C.

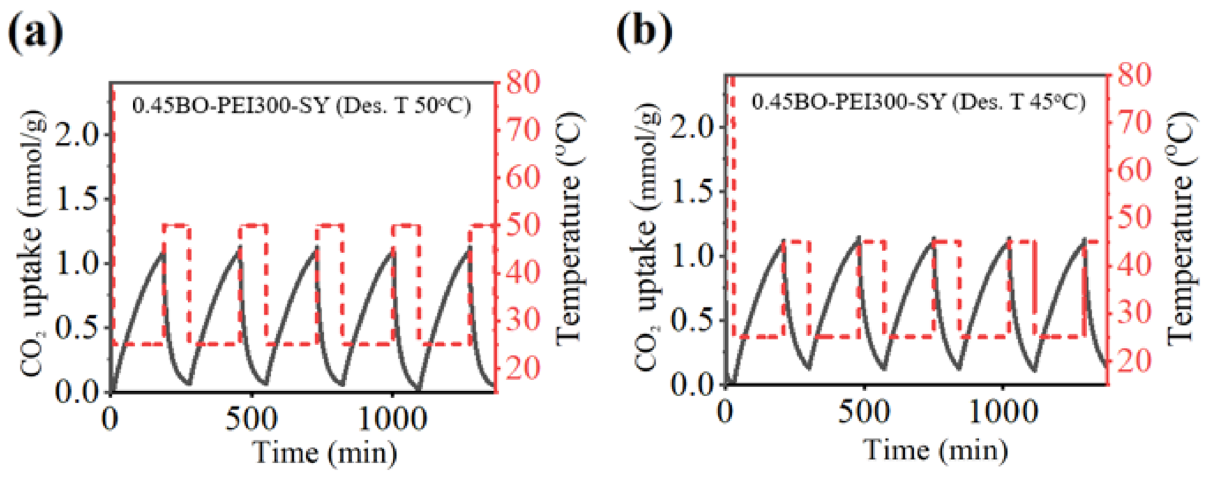

Figure S15. TGA adsorption–desorption curves of 0.45BO-PEI300-SY measured at desorption temperatures of (a) 50 °C and (b) 45 °C.

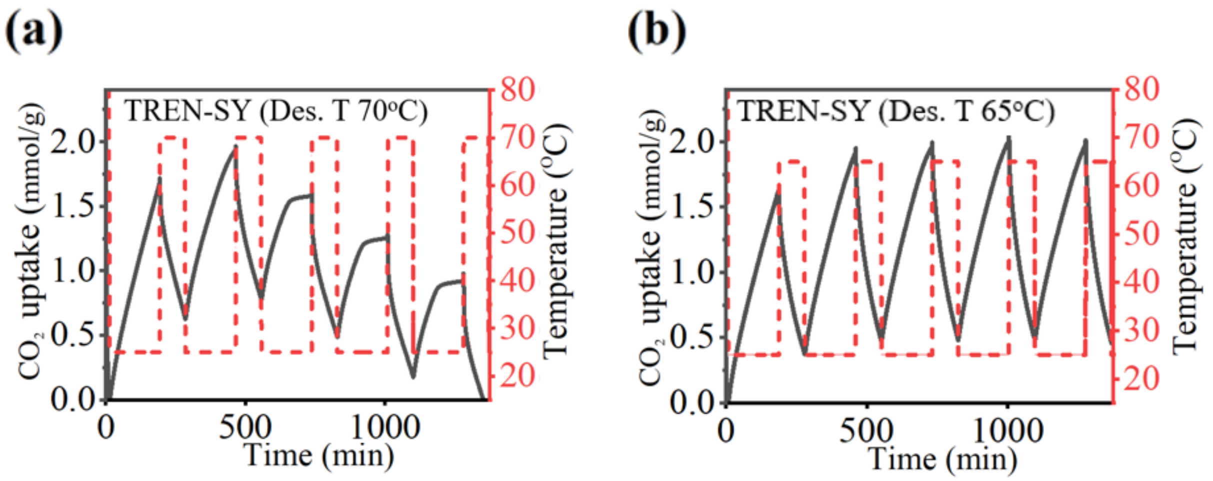

Figure S16. TGA adsorption–desorption curves of TREN-SY measured at desorption temperatures of (a) 70 °C and (b) 65 °C.

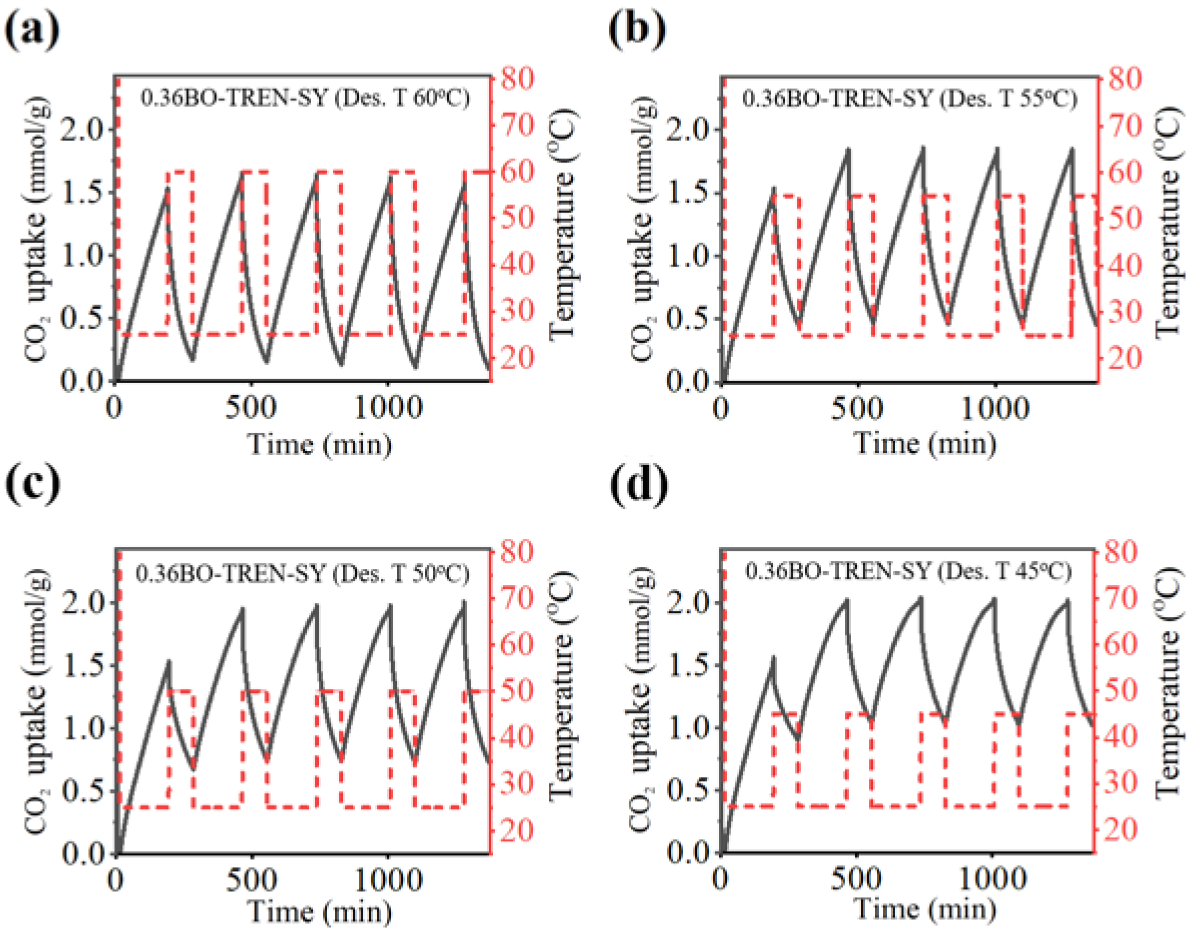

Figure S17. TGA adsorption–desorption curves of 0.36BO-TREN-SY measured at desorption temperatures of (a) 60 °C, (b) 55 °C, (c) 50 °C, and (d) 45 °C.

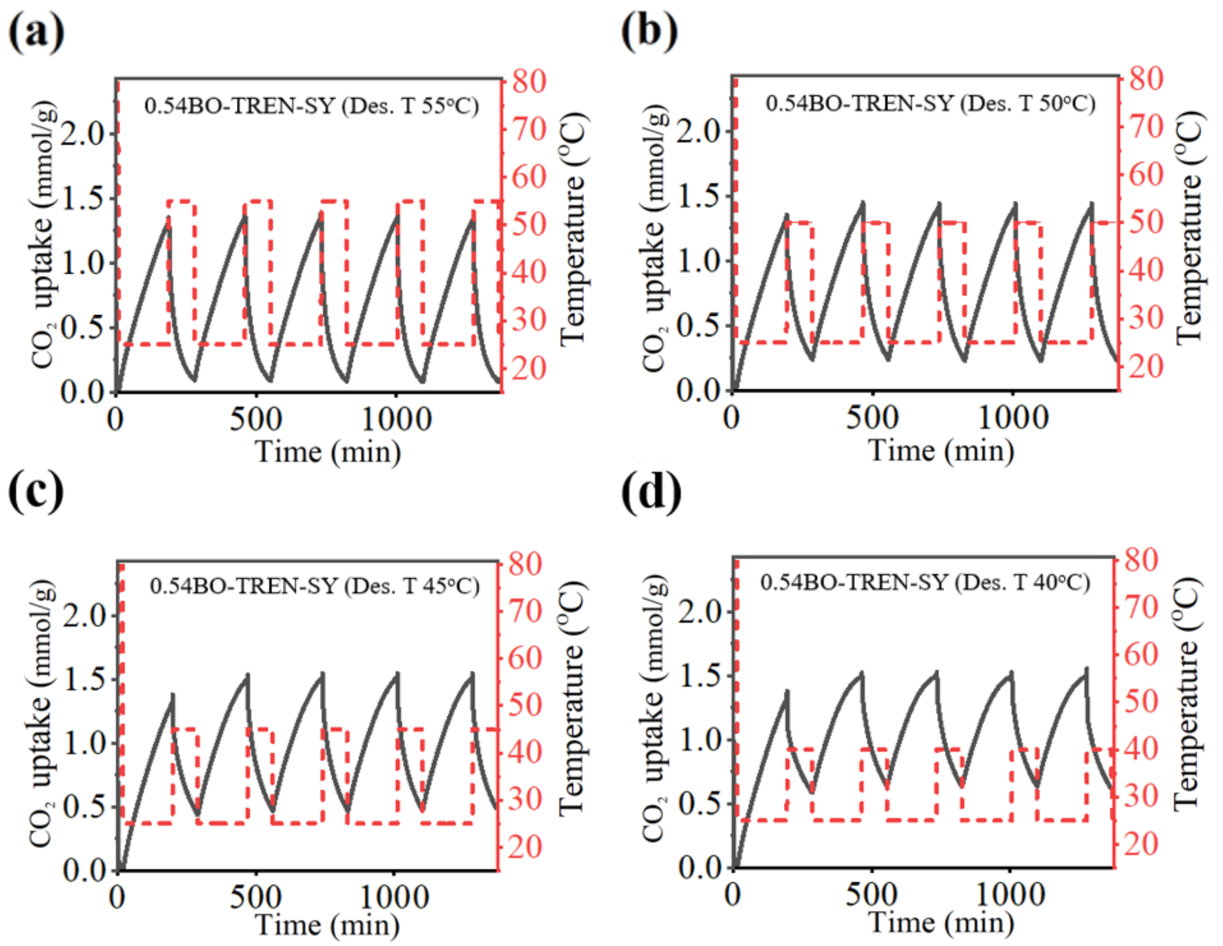

Figure S18. TGA adsorption–desorption curves of 0.54BO-TREN-SY measured at desorption temperatures of (a) 55 °C, (b) 50 °C, (c) 45 °C, and (d) 40 °C.

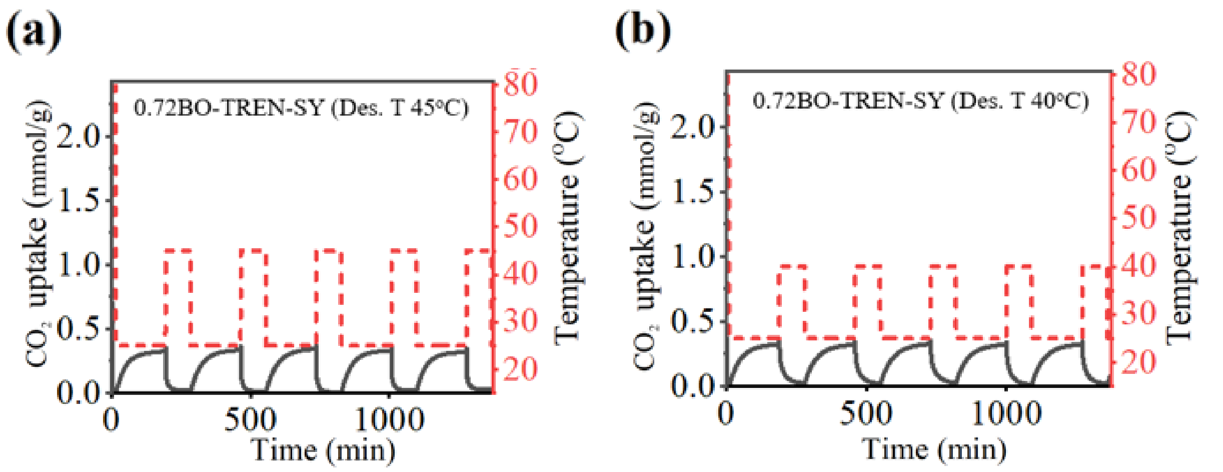

Figure S19. TGA adsorption–desorption curves of 0.72BO-TREN-SY measured at desorption temperatures of (a) 45 °C and (b) 40 °C.

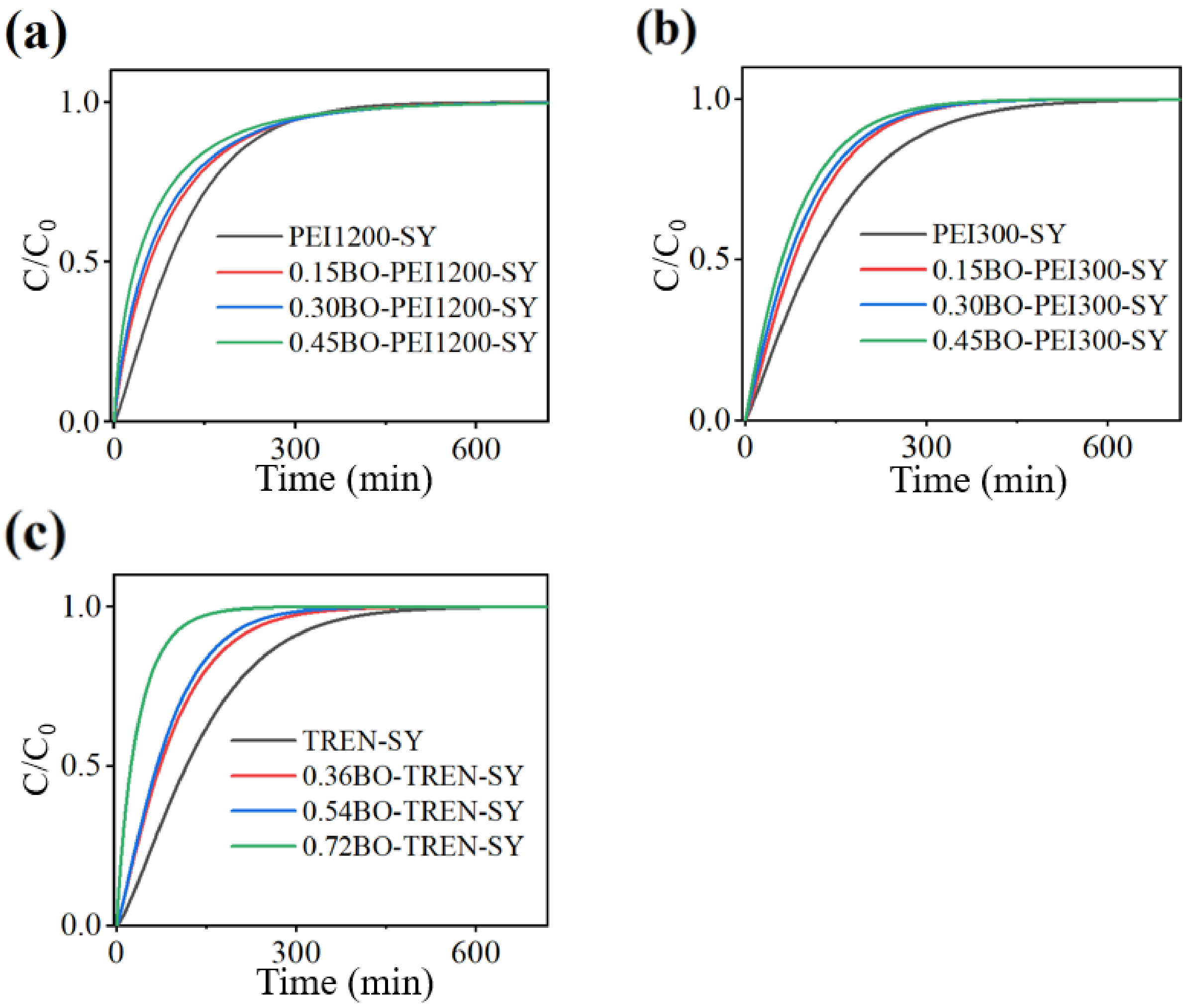

Figure S20. TGA curves of (a) PEI1200-SY and xBO-PEI1200-SY, (b) PEI300-SY and xBO-PEI300-SY, and (c) TREN-SY and xBO-TREN-SY for 12h adsorption at 25 °C under 400 ppm CO_2 with N_2 balance.

<Calculations of adsorption rate constants>

To determine the adsorption rate constant (k), the adsorption curves were fitted to the Avrami equation:

$$Q_t = Q_e[1 - \exp((-kt)^n)]$$

where n represents the kinetic order, k (min^{-1}) is the kinetic rate constant of the Avrami model, and Q_t and Q_e denote the adsorption capacities at time t and equilibrium, respectively.

Table S2. Kinetic parameters for CO_2 adsorption derived from the Avrami equation.

Sample	k (min^{-1})	N (-)	R^2 (-)
PEI1200-SY	0.0082	1.186	0.995
0.15BO-PEI1200-SY	0.0121	0.826	0.987
0.30BO-PEI1200-SY	0.0123	0.815	0.992
0.45BO-PEI1200-SY	0.0161	0.707	0.981
PEI300-SY	0.0067	1.178	0.987
0.15BO-PEI300-SY	0.0092	1.182	0.994
0.30BO-PEI300-SY	0.0101	1.107	0.999
0.45BO-PEI300-SY	0.0117	1.051	0.998
TREN-SY	0.0065	1.318	0.991
0.36BO-TREN-SY	0.0101	1.170	0.995
0.54BO-TREN-SY	0.0110	1.206	0.998
0.72BO-TREN-SY	0.0278	0.913	0.982

<Calculations of thermal energy requirements for TVSA system>

A simplified calculation of the thermal energy requirements for a temperature vacuum swing adsorption (TVSA) system was performed as follows:

The TVSA system consists of the four steps: (i) adsorption of external air, (ii) a pressure reduction combined with gradual heating to remove residual N_2 and other waste from the column, (iii) CO_2 desorption under vacuum, and (iv) re-introduction of air to initiate the subsequent adsorption cycle.

The thermal energy consumption (E_{th}, MJ/kg_{CO2}) for the TVSA system was established on the following equation [70]:

$$\mathbf{E_{th}} = \frac{\mathbf{m_{solids} C_{p,solids} (T_{des} - T_{amb})}}{(\mathbf{\Delta q_{CO_2} \times m_{solids} \times MW_{CO_2} \times 1000})} + \frac{(\mathbf{\Delta H_{CO_2} \times \Delta q_{CO_2} \times m_{solids}})}{(\mathbf{\Delta q_{CO_2} \times m_{solids} \times MW_{CO_2} \times 1000})}$$

Here, m_{solids} indicates the mass of adsorbent, $C_{p,solids}$ represents the heat capacity of the solid sorbent, ΔH_{CO_2} denotes the heat of adsorption, Δq_{CO_2} refers to the working capacity, MW_{CO2} is molecular weight of CO_2, and T_{des} and T_{amb} are desorption and ambient temperatures, respectively. The $C_{p,solids}$ and ΔH_{CO_2} for 0.30BO-PEI300-SY and 0.54BO-TREN-SY were adopted from APDES and TMCM-41, as they fall within the upper range reported for structurally similar amine-functionalized sorbents. The use of these relatively large values provides a conservative estimate of the thermal energy consumption.

Table S3. Parameters used for calculating thermal energy consumption.

Sample	$C_{p,solids}$ (J/°C)	ΔH_{CO2} (J/mol)	Δq_{CO2} (mmol/g)	T_{des} (°C)	T_{amb} (°C)
SIFSIX-18-Ni-β [a]	1000	52000	1.05	120	20
NbOFFIVE-1-Ni [a]	1000	50000	1.25	120	20
TPMS [a]	900	90000	0.4	120	20
TMCM-41 [a]	1000	90000	0.95	120	20
APDES [a]	2070	60000	1.1	120	20
0.30BO-PEI300-SY	2070	90000	1.09	45	25
0.54BO-TREN-SY	2070	90000	0.88	40	25

[a] : The parameters for these materials were taken from Ref. [18].

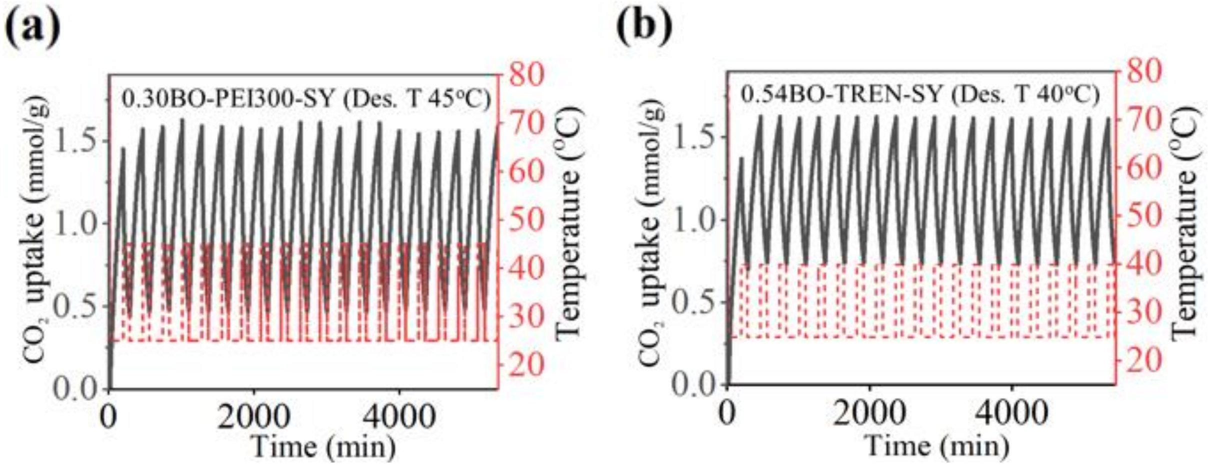

Figure S21. TGA adsorption–desorption curves of (a) 0.30BO-PEI300-SY measured at desorption temperature of 45 °C and (b) 0.54BO-TREN-SY measured at desorption temperature of 40 °C under the condition of CO_2 400 ppm/air during 20 cycles.

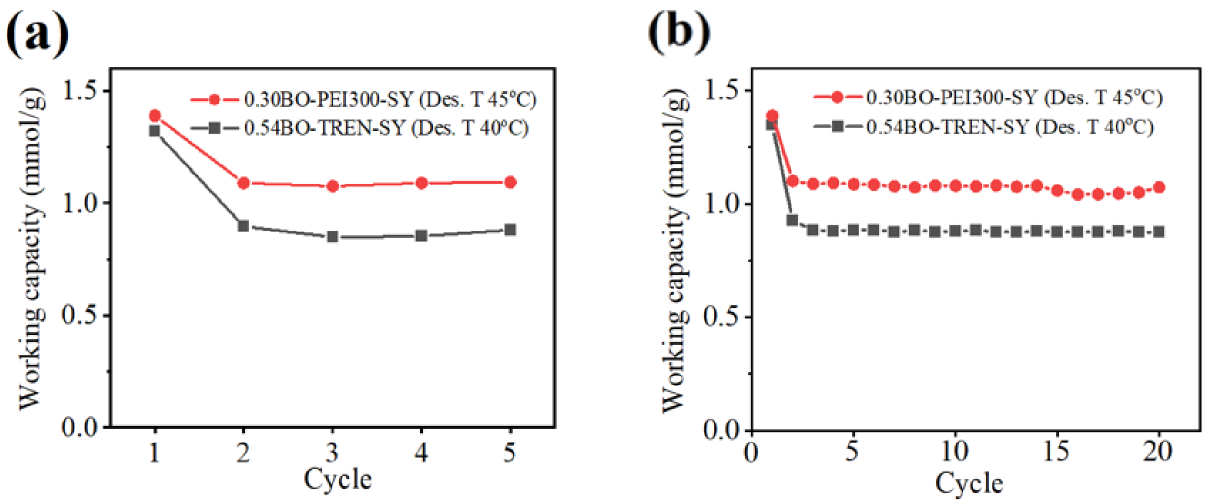

Figure S22. Working capacities of 0.30BO-PEI300-SY at a desorption temperature of 45 °C and 0.54BO-TREN-SY at 40 °C over consecutive adsorption–desorption TGA cycles. In (a), adsorption was conducted at 25 °C using a gas mixture of 400 ppm CO_2 and pure N_2 balance. In (b), adsorption was conducted at 25 °C using a gas mixture of 400 ppm CO_2 and air balance (79% N_2, 21% O_2).

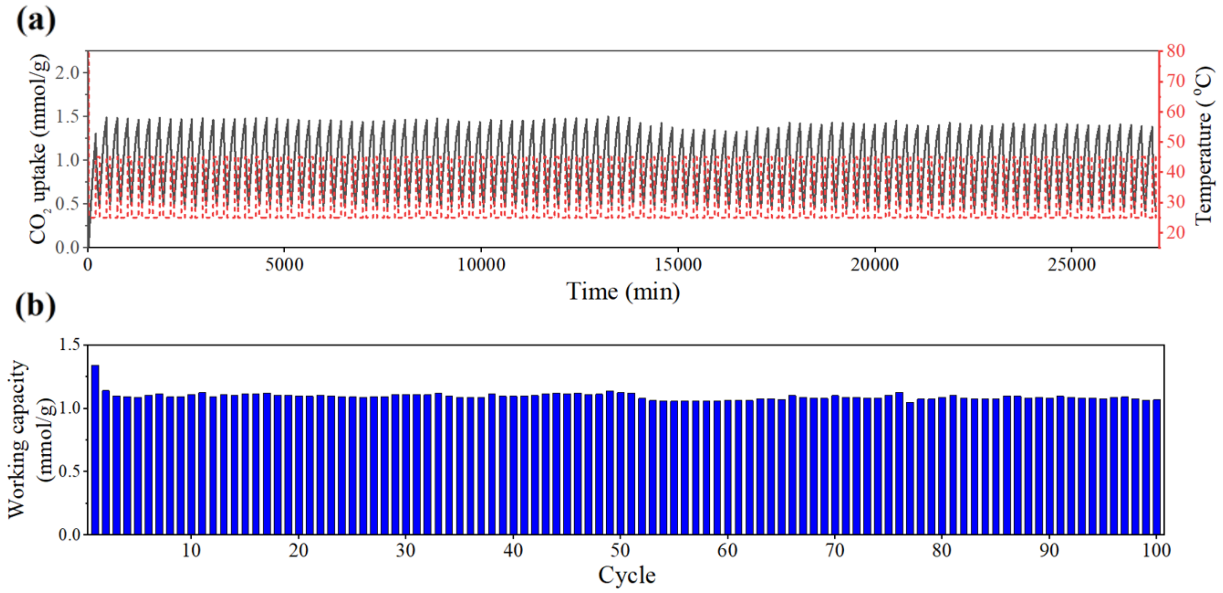

Figure S23. (a) TGA adsorption-desorption curves and (b) working capacity of 0.30BO-PEI300-SY over 100 adsorption-desorption cycles at a desorption temperature of 45 °C. Adsorption was conducted at 25 °C using a gas mixture of 400 ppm CO_2 in air (79% N_2, 21% O_2).

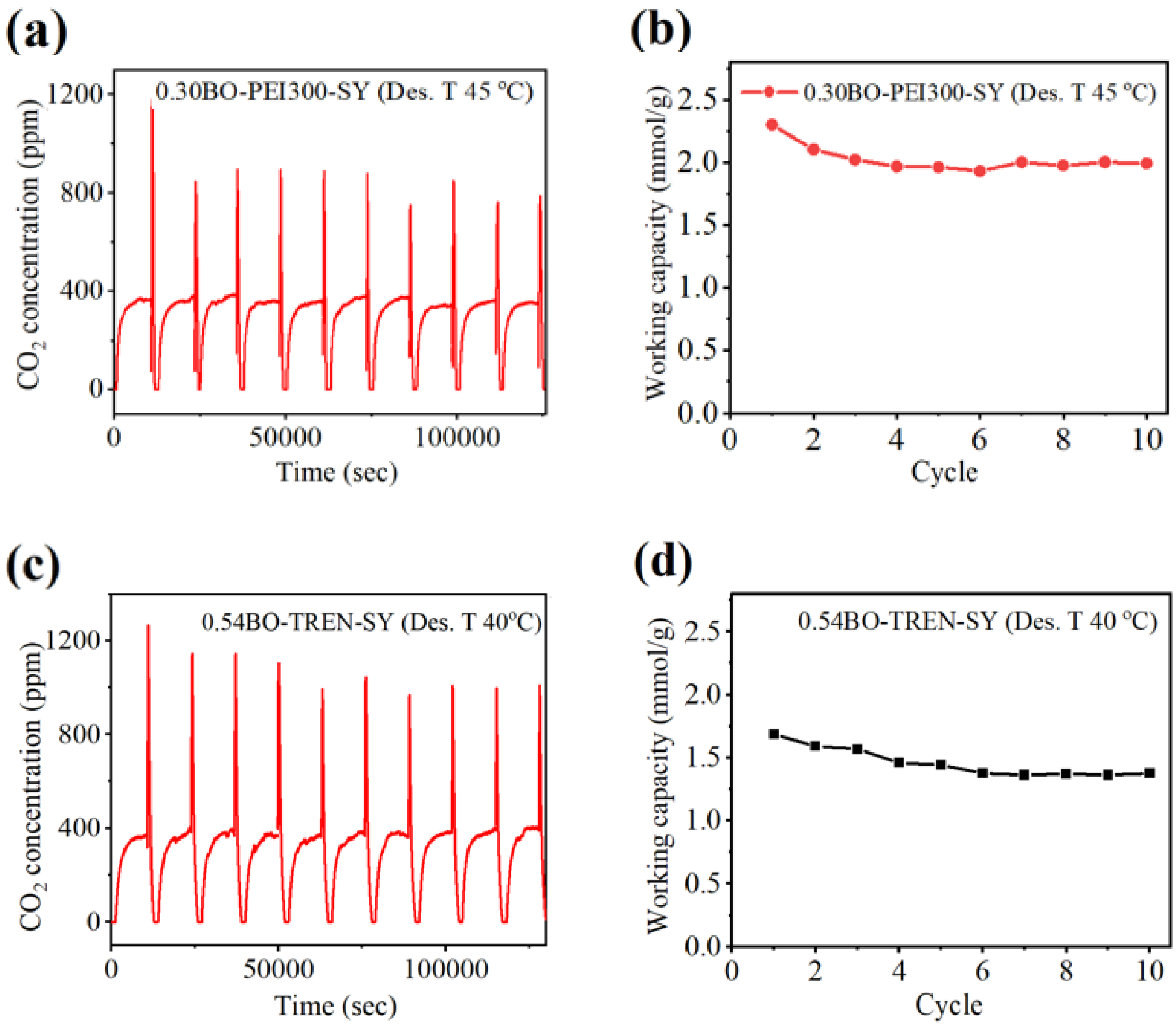

Figure S24. (a) CO_2 concentration and (b) working capacity at adsorption–desorption breakthrough experiment of 0.30BO-PEI300-SY. (c) CO_2 concentration and (d) working capacity at adsorption–desorption breakthrough experiment of 0.54BO-TREN-SY. Experimental conditions: 50% humidity, CO_2 400 ppm/N_2, 10 cycles.

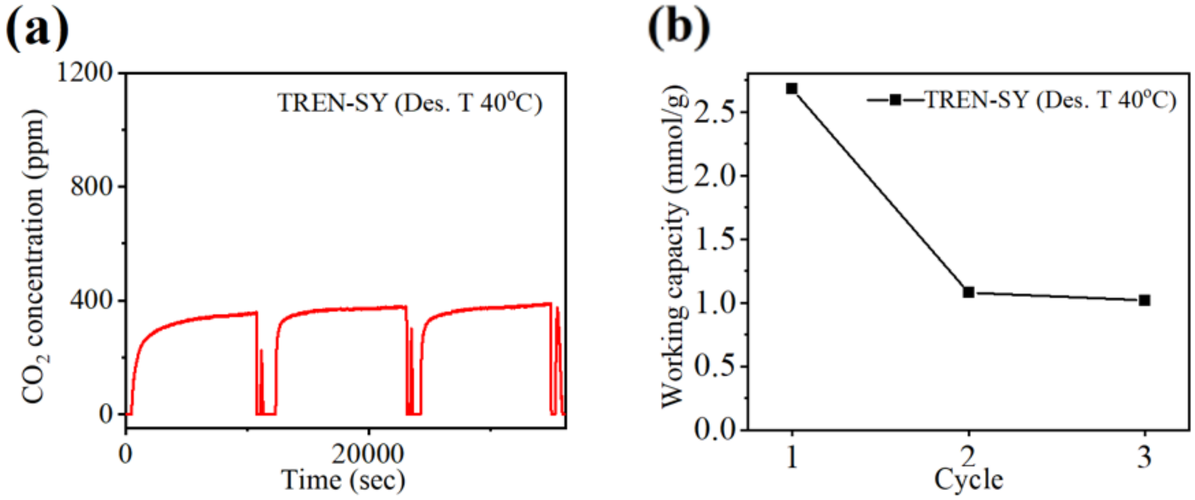

Figure S25. (a) CO_2 concentration and (b) working capacity at adsorption–desorption breakthrough experiment of TREN-SY under 50% humidity, CO_2 400 ppm/N_2, and 3 cycles.

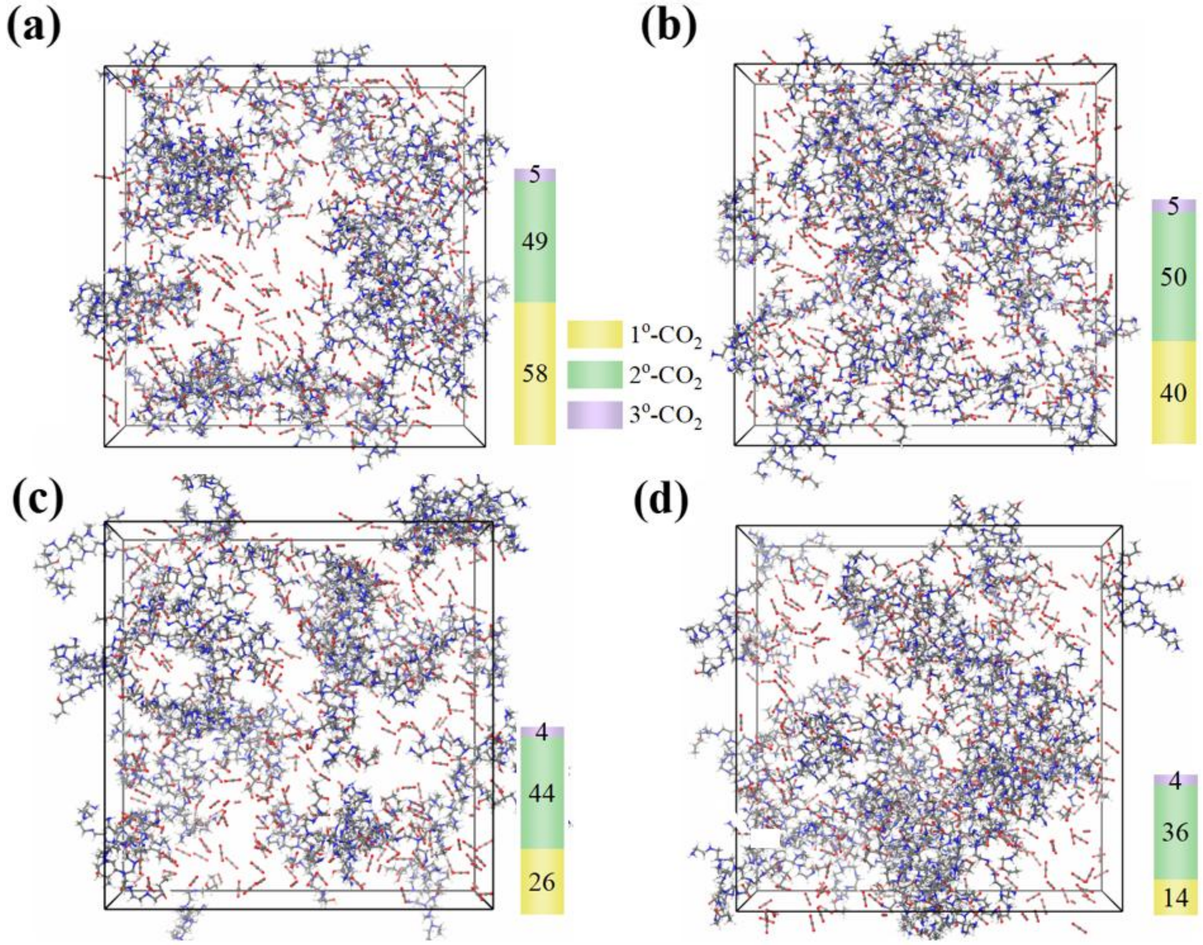

Figure S26. Snapshots of CO_2 adsorption on (a) pristine PEI1200, (b) 0.15BO-PEI1200, (c) 0.30BO-PEI1200, and (d) 0.45BO-PEI1200 obtained from MD simulations. The accompanying bar plot represents the number of CO_2 molecules adsorbed on 1°, 2°, and 3° amines. N_2 molecules are omitted for clarity.

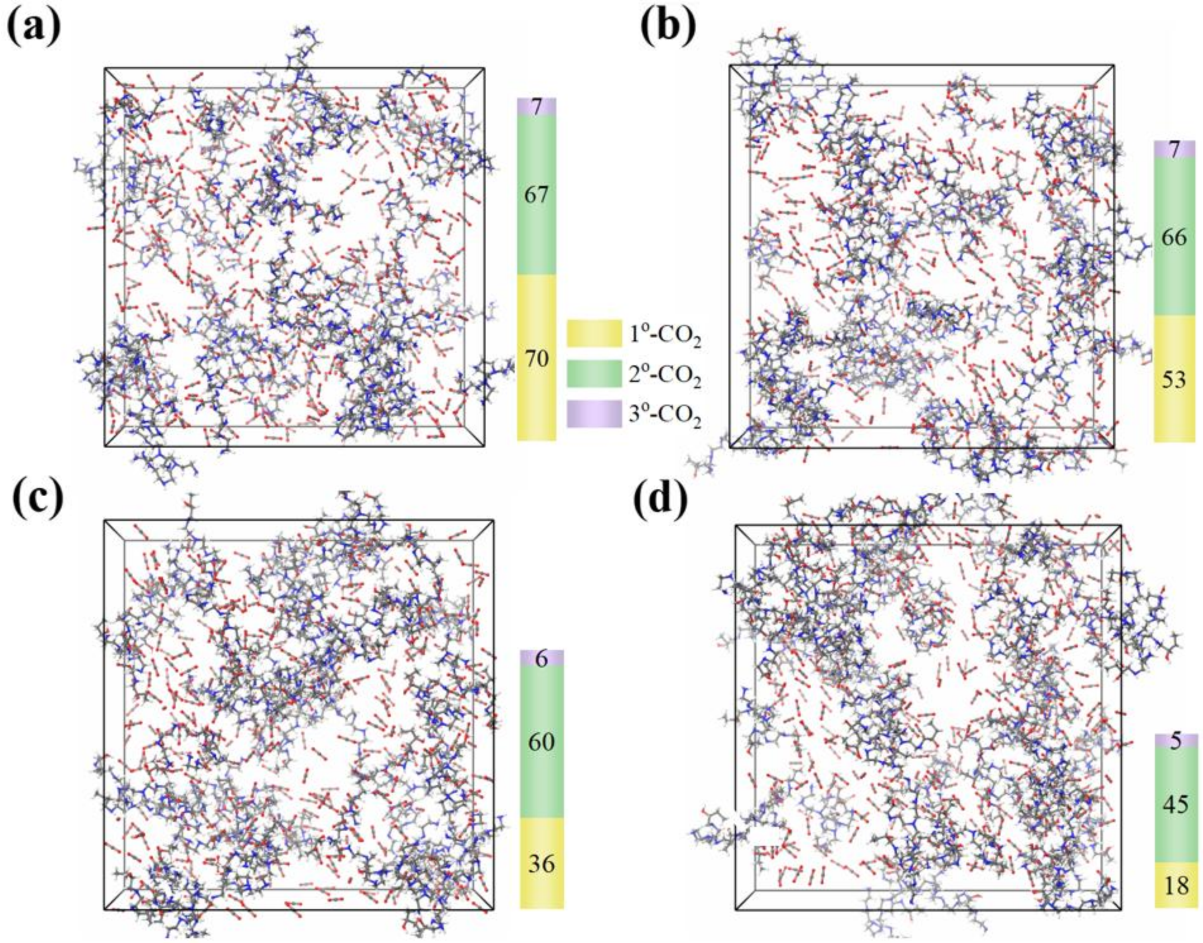

Figure S27. Snapshots of CO_2 adsorption on (a) pristine PEI300, (b) 0.15BO-PEI300, (c) 0.30BO-PEI300, and (d) 0.45BO-PEI300 obtained from molecular dynamics simulations. The accompanying bar plot represents the number of CO_2 molecules adsorbed on 1°, 2°, and 3° amines. N_2 molecules are omitted for clarity.

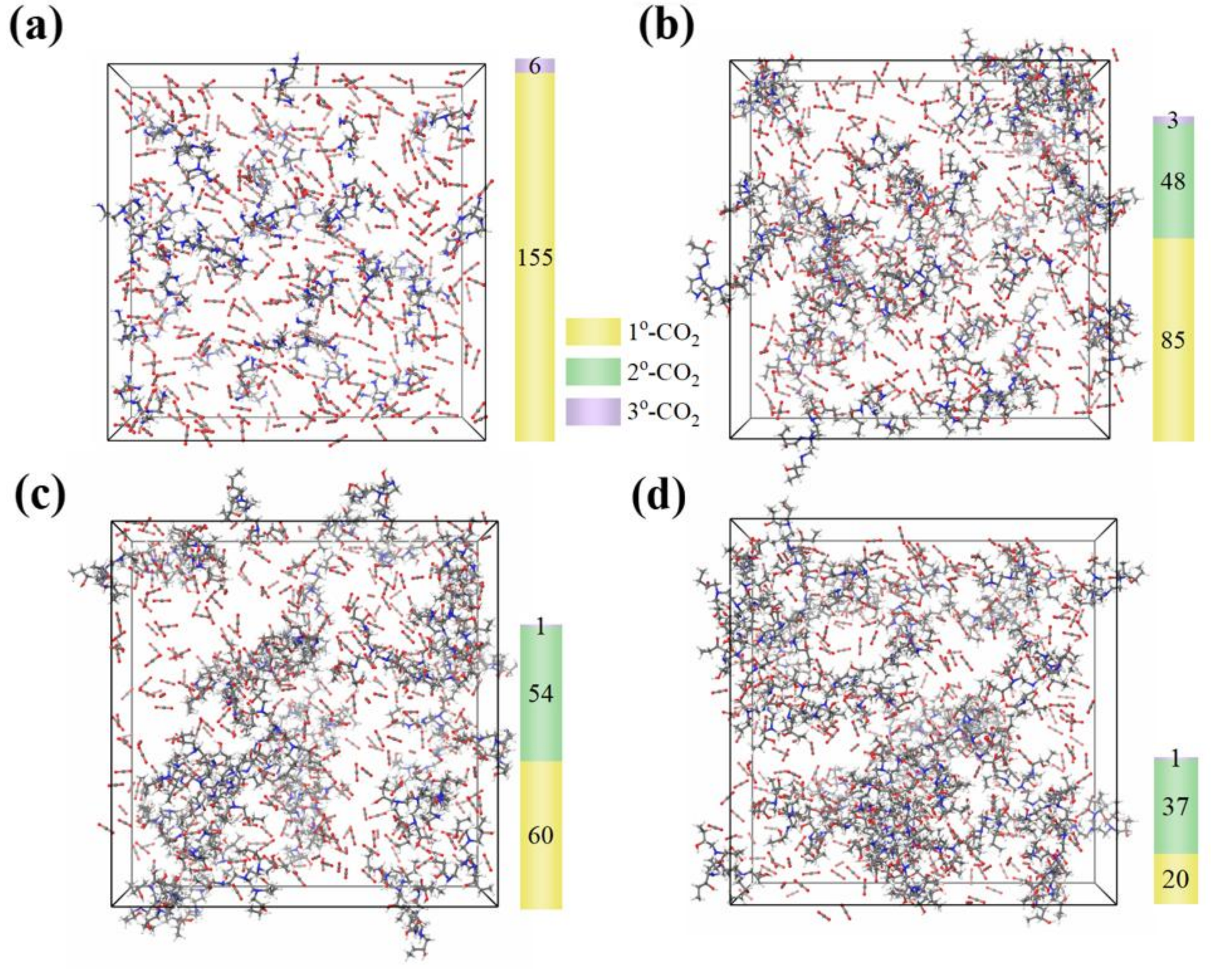

Figure S28. Snapshots of CO_2 adsorption on (a) pristine TREN, (b) 0.36BO-TREN, (c) 0.54BO-TREN, and (d) 0.72BO-TREN obtained from molecular dynamics simulations. The accompanying bar plot represents the number of CO_2 molecules adsorbed on 1°, 2°, and 3° amines. N_2 molecules are omitted for clarity.

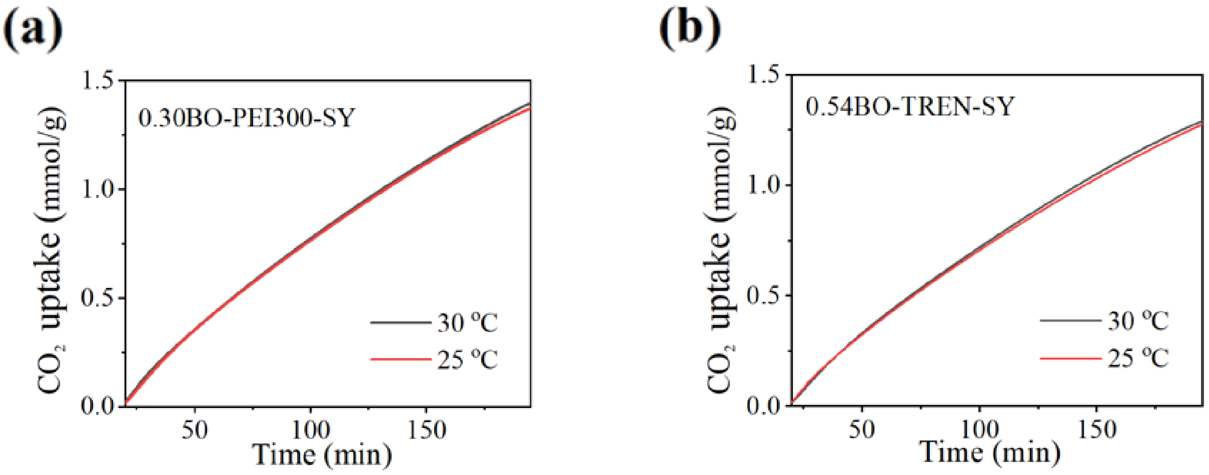

Figure S29. TGA adsorption curves of (a) 0.30BO-PEI300-SY and (b) 0.54BO-TREN-SY measured at adsorption temperatures of 25 °C and 30 °C under dry 400 ppm CO_2/N_2 conditions.

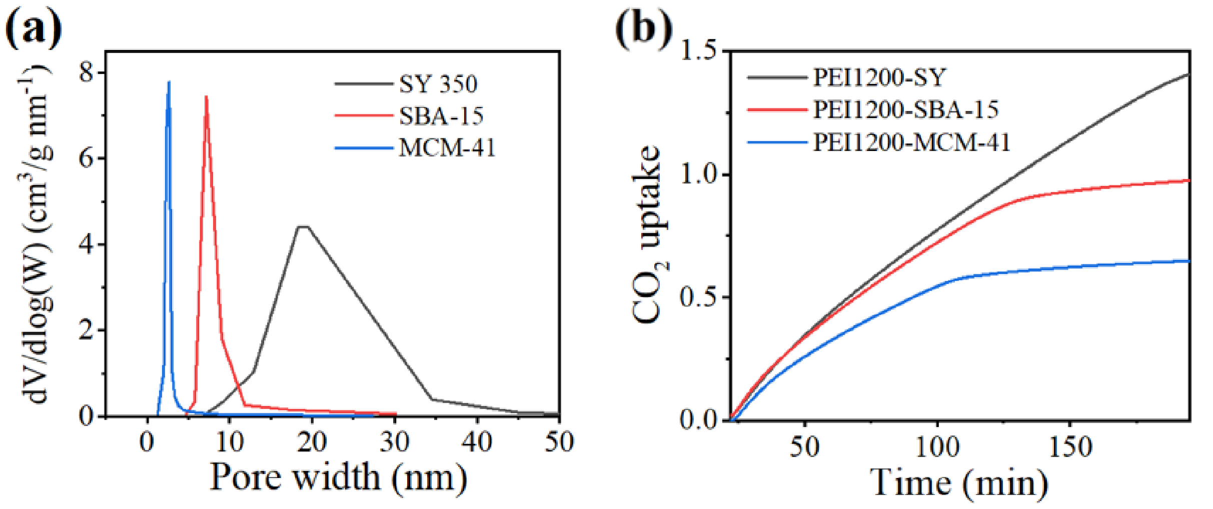

Figure S30. (a) Pore size distributions of three different mesoporous silicas (SY 350, SBA-15, and MCM-41). (b) TGA adsorption curves of PEI1200-impregnated silicas under dry 400 ppm CO_2/N_2 conditions at 25 °C. The amine was impregnated into the three mesoporous silica supports using a fixed 1:1 mass ratio to ensure consistency across all samples.

Table S4. Number of CO_2 molecules adsorbed on 1°, 2°, and 3° amines in pristine Amine and xBO-Amine structures, as calculated by MD simulations.

Molecule	1°-CO_2	2°-CO_2	3°-CO_2	Total
PEI1200	58	49	5	112
0.15BO-PEI1200	40	50	5	95
0.30BO-PEI1200	26	44	4	74
0.45BO-PEI1200	14	36	4	54
PEI300	70	67	7	144
0.15BO-PEI300	53	66	7	126
0.30BO-PEI300	36	60	6	102
0.45BO-PEI300	18	45	5	68
TREN	155	0	6	161
0.36BO-TREN	85	48	3	136
0.54BO-TREN	60	54	1	115
0.72BO-TREN	20	37	1	58

Table S5. Comparison of WC_{cyclic} values with benchmark DAC sorbents under dry conditions.

Sorbent	Conditions					WC_{cyclic} (mmol g^{-1})	Cycling stability (cycles) [c]	Ref.
	Adsorption		Desorption					
	Time (h)	Temp. (°C)	Time (h)	Temp. (°C)	Carrier gas			
0.30BO-PEI300-SY	3	25	1.5	45	N_2	1.09 [a]	0.09 % loss (20-100 cycles)	This work
0.54BO-TREN-SY	3	25	1.5	40	N_2	0.88 [a]	0.4 % loss (10-20 cycles)	This work
40TEPA-10PEG/SiO_2	7	30	1.5	90	N_2	2.13 [a]	2.4 % loss (0-20 cycles)	[1]
Ph-3-ED/SBA-15	3	35	0.16	90	He	1.4 [a]	no loss (0-20 cycles)	[2]
TEPA/SBA-15	1	25	0.25	90	N_2	1.8 [a]	0.6 % loss (5-10 cycles)	[3]
Na[TFPA]-F-COF	2.5	25	-	90	He	1.86 [b]	1.6 % loss (3-5 cycles)	[4]
MIL-101(Cr)-PEI-PEG	2	25	1	110	N_2	1.65 [a]	1.8 % loss (3-5 cycles)	[5]
Cr-MIL-101-SO_3 H-TETA	-	20	1	80	Vacuum	1.12 [a]	2.6 % loss (0-10 cycles)	[6]
MOF-74-mmen-2	1	25	0.5	150	N_2/O_2	1.05 [a]	1.1 % loss (0-10 cycles)	[7]
$Zn(ZnOH)_4(bibta)_3$	4	25	1	100	N_2/O_2	1.32 [a]	5.3 % loss (3-5 cycles)	[8]

[a]: TGA conditions; [b]: breakthrough conditions; [c]: the later cycles used for calculating WC_{cyclic}.

Table S6. Comparison of WC_{cyclic} values with benchmark DAC sorbents under humid breakthrough conditions.

Sorbent	Conditions						WC_{cyclic} (mmol g^{-1})	Cycling stability (cycles) [a]	Ref.
	Adsorption			Desorption					
	Time (h)	RH (%)	Temp. (°C)	Time (h)	Temp. (°C)	Carrier gas			
0.30BO-PEI300-SY	3	50	30	0.5	45	He	1.99	no loss (5-10 cycles)	This work
0.54BO-TREN-SY	3	50	30	0.5	40	He	1.37	3.4 % loss (5-10 cycles)	This work
PEHA-PO-1-2/50S	3	50	25	0.25	50	N_2	1.22	0.81 % loss (0-10 cycles)	[9]
S/D1/P1	1	50	25	0.5	80	N_2	1.4	1.0 % loss (5-10 cycles)	[10]
PEI/PME(40)	4.1	64	25	-	90	N_2	2.92	1.0 % loss (10-20 cycles)	[11]
PEI40/FAU300	4	60	25	1	80	Ar	1.0	7.0% loss (5-10 cycles)	[12]
IN-TRI-LDH	0.67	20	25	0.08	70	N_2	0.88	2.5 % loss (20-50 cycles)	[13]
COF-999	3	50	25	1.67	60	N_2/O_2	2.02	no loss (0-10 cycles)	[14]
COF-709	6	75	25	1	60	N_2	1.23	1.2 % loss (0-10 cycles)	[15]
MOF-808-Lys	5.83	50	25	1	140	N_2/O_2	0.74	8.1 % loss (0-10 cycles)	[16]
PEI@H-SiO_2	12	19	30	-	110	He	3.36	6.7 % loss (3-5 cycles)	[17]

[a]: the later cycles used for calculating WC_{cyclic}.

References

[1] Z. Yang, Y. Zhou, H. Cui, Z. Cheng, Z. Zhou, *Frontiers of Chemical Science Engineering.* **2025**, *19* (2), 9. https://doi.org/10.1007/s11705-024-2512-3.

[2] D. R. Kumar, C. Rosu, A. R. Sujan, M. A. Sakwa-Novak, E. W. Ping, C. Jones, *ACS Sustainable Chemistry Engineering.* **2020**, *8* (29), 10971. https://doi.org/10.1021/acssuschemeng.0c03706.

[3] Y. Miao, Z. He, X. Zhu, D. Izikowitz, J. Li, *Chemical Engineering Journal.* **2021**, *426*, 131875. https://doi.org/10.1016/j.cej.2021.131875.

[4] L. Qiu, M. Lei, C. Wang, J. Hu, L. He, A. S. Ivanov, D. e. Jiang, H. Lin, I. Popovs, Y. Song, *Small.* **2024**, *20* (36), 2401798. https://doi.org/10.1002/smll.202401798.

[5] K. Jiang, J. Yang, X. Liu, Y. Tong, J. Liu, J. Gu, *Chemical Engineering Journal.* **2025**, *510*, 161643. https://doi.org/10.1016/j.cej.2025.161643.

[6] H. Li, K. Wang, D. Feng, Y. P. Chen, W. Verdegaal, H. C. Zhou, *ChemSusChem.* **2016**, *9* (19), 2832. https://doi.org/10.1002/cssc.201600768.

[7] T. M. McDonald, W. R. Lee, J. A. Mason, B. M. Wiers, C. S. Hong, J. R. Long, *Journal of the American Chemical Society.* **2012**, *134* (16), 7056. https://doi.org/10.1021/ja300034j.

[8] C. E. Bien, K. K. Chen, S.-C. Chien, B. R. Reiner, L.-C. Lin, C. R. Wade, W. W. Ho, *Journal of the American Chemical Society.* 2018, *140* (40), 12662. https://doi.org/10.1021/jacs.8b06109.

[9] A. Goeppert, H. Zhang, R. Sen, H. Dang, G. S. J. C. Prakash, *ChemSusChem.* **2019**, *12* (8), 1712. https://doi.org/10.1002/cssc.201802978.

[10] Y. Miao, Y. Wang, B. Ge, Z. He, X. Zhu, J. Li, S. Liu, L. Yu, *Advanced Science.* **2023**, *10* (16), 2207253. https://doi.org/10.1002/advs.202207253.

[11] A. Sayari, Q. Liu, P. Mishra, *ChemSusChem.* **2016**, *9* (19), 2796. https://doi.org/10.1002/cssc.201600834.

[12] R. Kumar, S. Ohtani, N. Tsunoji, *Microporous and Mesoporous Materials.* **2023**, *360*, 112714. https://doi.org/10.1016/j.micromeso.2023.112714.

[13] B. Ge, C. Chen, Y. Xu, S. Roberts, M. Zhang, Q. Shao, D. O'Hare, X. Zhu, *Chemical Engineering Journal.* **2024**, *500*, 156782. https://doi.org/10.1016/j.cej.2024.156782.

[14] Z. Zhou, T. Ma, H. Zhang, S. Chheda, H. Li, K. Wang, S. Ehrling, R. Giovine, C. Li, A. H. Alawadhi, *Nature.* **2024**, *635* (8037), 96. https://doi.org/10.1038/s41586-024-08080-x.

[15] H. Li, Z. Zhou, T. Ma, K. Wang, H. Zhang, A. H. Alawadhi, O. M. Yaghi, *Journal of*

the American Chemical Society. **2024**, *146* (51), 35486. https://doi.org/10.1021/jacs.4c14971.

[16] O. I.-F. Chen, C.-H. Liu, K. Wang, E. Borrego-Marin, H. Li, A. H. Alawadhi, J. A. Navarro, O. M. Yaghi, *Journal of the American Chemical Society.* **2024**, *146* (4), 2835. https://doi.org/10.1021/jacs.3c14125.

[17] H. T. Kwon, M. A. Sakwa-Novak, S. H. Pang, A. R. Sujan, E. W. Ping, C. W. Jones, *Chemistry of materials.* **2019**, *31* (14), 5229. https://doi.org/10.1021/acs.chemmater.9b01474.

[18] B.M. Balasubramaniam, P-T Thierry, S. Lethier, V. Pugnet, P. Llewellyn, A. Rajendran. *Chemical Engineering Journal.* 2024, 485, 149568. https://doi.org/10.1016/j.cej.2024.14956
